# Review of childhood genetic nephrolithiasis and nephrocalcinosis

**DOI:** 10.3389/fgene.2024.1381174

**Published:** 2024-03-28

**Authors:** Ashley M. Gefen, Joshua J. Zaritsky

**Affiliations:** Phoenix Children’s Hospital, Department of Pediatrics, Division of Nephrology, Phoenix, AZ, United States

**Keywords:** children, genetic kidney disease, kidney stones, nephrolithiasis, nephrocalcinosis

## Abstract

Nephrolithiasis (NL) is a common condition worldwide. The incidence of NL and nephrocalcinosis (NC) has been increasing, along with their associated morbidity and economic burden. The etiology of NL and NC is multifactorial and includes both environmental components and genetic components, with multiple studies showing high heritability. Causative gene variants have been detected in up to 32% of children with NL and NC. Children with NL and NC are genotypically heterogenous, but often phenotypically relatively homogenous, and there are subsequently little data on the predictors of genetic childhood NL and NC. Most genetic diseases associated with NL and NC are secondary to hypercalciuria, including those secondary to hypercalcemia, renal phosphate wasting, renal magnesium wasting, distal renal tubular acidosis (RTA), proximal tubulopathies, mixed or variable tubulopathies, Bartter syndrome, hyperaldosteronism and pseudohyperaldosteronism, and hyperparathyroidism and hypoparathyroidism. The remaining minority of genetic diseases associated with NL and NC are secondary to hyperoxaluria, cystinuria, hyperuricosuria, xanthinuria, other metabolic disorders, and multifactorial etiologies. Genome-wide association studies (GWAS) in adults have identified multiple polygenic traits associated with NL and NC, often involving genes that are involved in calcium, phosphorus, magnesium, and vitamin D homeostasis. Compared to adults, there is a relative paucity of studies in children with NL and NC. This review aims to focus on the genetic component of NL and NC in children.

## 1 Introduction

In the United States, nephrolithiasis (NL) is relatively common, affecting approximately 1 in 11 adults (Scales et al., 2012). A study of 12.7 million children in the United States noted a rate of NL of 54.1 cases per 100,000 person-years in 2016 (Ward et al., 2019). Multiple studies have noted that the incidence of NL and nephrocalcinosis (NC) has increased over the past decade, especially in adolescents and female children (Novak et al., 2009; Dwyer et al., 2012; Ward et al., 2019; Li et al., 2020). There is high morbidity associated with NL and NC, including an increased risk of chronic kidney disease (CKD) and kidney failure (Zhe and Hang, 2017). In addition, the economic burden associated with treatment is high with an annual expenditure of over $10 billion in the United States based on data from 2006 (Economic Impact of Urologic Disease, 2012). In adults, obesity, metabolic syndrome, hypertension, and diabetes have been associated with NL (Taylor et al., 2005; Lieske et al., 2006). The etiology of NL and NC is multifactorial and includes both environmental components and genetic components. Compared to adults, there is a relative paucity of studies in children with NL and NC. This review aims to focus on the genetic component of NL and NC in children.

## 2 Heritability of nephrolithiasis and nephrocalcinosis

Twin studies have illustrated 56% heritability for NL with a significantly higher frequency in female twins compared to male twins (Goldfarb et al., 2005; Goldfarb et al., 2018). Twin studies and large population studies have also shown heritability of serum and urine electrolytes, with that of serum calcium and 24-h urine calcium ranging from 33% to 37% and 44%–52%, respectively (Hunter et al., 2002; Moulin et al., 2017). Heritability of 80% has also been noted for serum 25-hydroxy-vitamin D levels (Wjst et al., 2007).

## 3 Genetic causes of nephrolithiasis and nephrocalcinosis in children

Causative gene variants have been detected in 11.5%–31.9% of children with NL and NC using high-throughput multiplex PCR with next-generation exon sequencing (Halbritter et al., 2015; Braun et al., 2016; Gefen et al., 2023). Causative variants have been detected in 29.4% of children with NL and NC using whole exome sequencing of 30 genes associated with monogenic NL and NC and in 32.5% of children with NL using whole exome sequencing of 38 genes associated with monogenic NL in a pediatric Chinese cohort.

Children with NL and NC are genotypically heterogenous, but often phenotypically relatively homogenous, and there are subsequently little data on the predictors of genetic childhood NL and NC. The discordance between phenotype and genotype is well illustrated by a study that showed that 23.9% and 7.3% of children with suspected Dent disease and primary hyperoxaluria (PH), respectively, based on their phenotype were found to have disease causing variants in unrelated genes (Cogal et al., 2021). Regarding identified risk factors for genetic NL in children, a Chinese study found the following: positive family history, consanguinity, younger age at onset, presence of concurrent NC, and CKD (Zhao et al., 2022). In contrast, an American study of children with NL and NC found that the only factor predictive of genetic disease was low serum bicarbonate (Gefen et al., 2023).

Given the high diagnostic yield of genetic testing in this population as well as the genetic heterogeneity, phenotypic homogeneity, and lack of well-established predictors of genetic NL and NC, performing targeted genetic testing broadly for children with NL and NC has been suggested. Genetic testing has become more readily available in recent years with high throughput exon sequencing with whole-exome sequencing or exon panels. Genetic testing may provide clinically meaningful information and affect long-term outcomes. According to multiple studies in children with kidney diseases including NL and NC, genetic testing has the power to alter clinical management and surveillance in >40% of cases (Halbritter et al., 2015; Amlie-Wolf et al., 2021; Jayasinghe et al., 2021).

### 3.1 Conditions with hypercalciuria

Most genetic diseases associated with NL and NC are secondary to hypercalciuria, including those secondary to hypercalcemia, renal phosphate wasting, renal magnesium wasting, distal renal tubular acidosis (RTA), proximal tubulopathies, mixed or variable tubulopathies, Bartter syndrome, hyperaldosteronism and pseudohyperaldosteronism, hyperparathyroidism and hypoparathyroidism, and other causes. Each of these categories will be expanded on in the following subsections.

#### 3.1.1 Hypercalcemia and hypocalcemia

Table 1 contains a list of genetic causes of primary hypercalcemia and hypocalcemia with hypercalciuria and NL and/or NC. These conditions are due to variants in *CASR* and *CYP24A1* and will be discussed in greater detail below.

**TABLE 1:** Genetic causes of primary hypercalcemia and hypocalcemia with hypercalciuria, nephrolithiasis and/or nephrocalcinosis.

Gene	Gene product	Phenotype	OMIM phenotype number	Inheritance	Description
*CASR*	Calcium-sensing receptor (PT, TAL, DCT, CD)	AD hypocalcemia/AD hypocalcemia with Bartter syndrome (Pearce et al., 1996; Vargas-Poussou et al., 2002)	601198	AD	Hypocalcemia, hypoparathyroidism, hypercalciuria, associated with NC, NL. Some may have features of Bartter syndrome such as impaired sodium chloride reabsorption in TAL, hypokalemic metabolic alkalosis, hyperreninemia and/or hyperaldosteronism
		Neonatal hyperparathyroidism (Lila et al., 2012; El Allali et al., 2021)	239200	AD/AR	Severe hypercalcemia and hyperparathyroidism associated with NC, NL
		Hypocalciuric hypercalcemia type 1 (Carling et al., 2000)	145980	AD	Lifelong mild to moderate hypercalcemia, inappropriate hypocalciuria, normal or mildly elevated circulating PTH, typically hypermagnesemia, but atypical presentations have been described with severe hypercalcemia with hypercalciuria, with or without NL or NC
*CYP24A1*	Cytochrome P450 family 24 subfamily A member 1/1,25-dihydroxy-vitamin D3 24-hydroxylase (PT)	Infantile hypercalcemia 1 (Schlingmann et al., 2011)	143880	AR	Accumulation of 1,25-dihydroxyvitamin D3. With severe hypercalcemia, failure to thrive, vomiting, dehydration, NCNL.
		CYP24A1 carrier (Carpenter, 2017)	N/A	Carrier	May have increased risk of NC, NL

AD, autosomal dominant; AR, autosomal recessive; CD, collecting duct; DCT, distal convoluted tubule; NC, nephrocalcinosis; NL, nephrolithiasis; PT, proximal tubule; TAL, thick ascending loop of Henle.

Supplementary Table S1 contains a list of genetic causes of secondary hypercalcemia and hypocalcemia with hypercalciuria and NL and/or NC. Conditions associated with secondary hypercalcemia include Williams-Beuren syndrome (7p11.23, autosomal dominant [AD] inheritance, OMIM phenotype number 194050), Oculoskeletodental syndrome (*PIK32CA* gene, autosomal recessive [AR] inheritance, OMIM phenotype number 618440) (Tiosano et al., 2019), Blue diaper syndrome (possibly *PCSK1* gene, AR *versus* X-linked recessive [XLR] inheritance, OMIM phenotype number 211000) (Drummond et al., 1964; Distelmaier et al., 2024), Congenital surcease-isomaltase deficiency (*SI* gene, AR inheritance, OMIM phenotype number 222900) (Starnes and Welsh, 1970; Belmont et al., 2002), and Glucose/galactose malabsorption (*SLC5A1* gene, AR inheritance, OMIM phenotype number 606824) (Soylu et al., 2008).

##### 3.1.1.1 CASR gene

**FIGURE 1:**
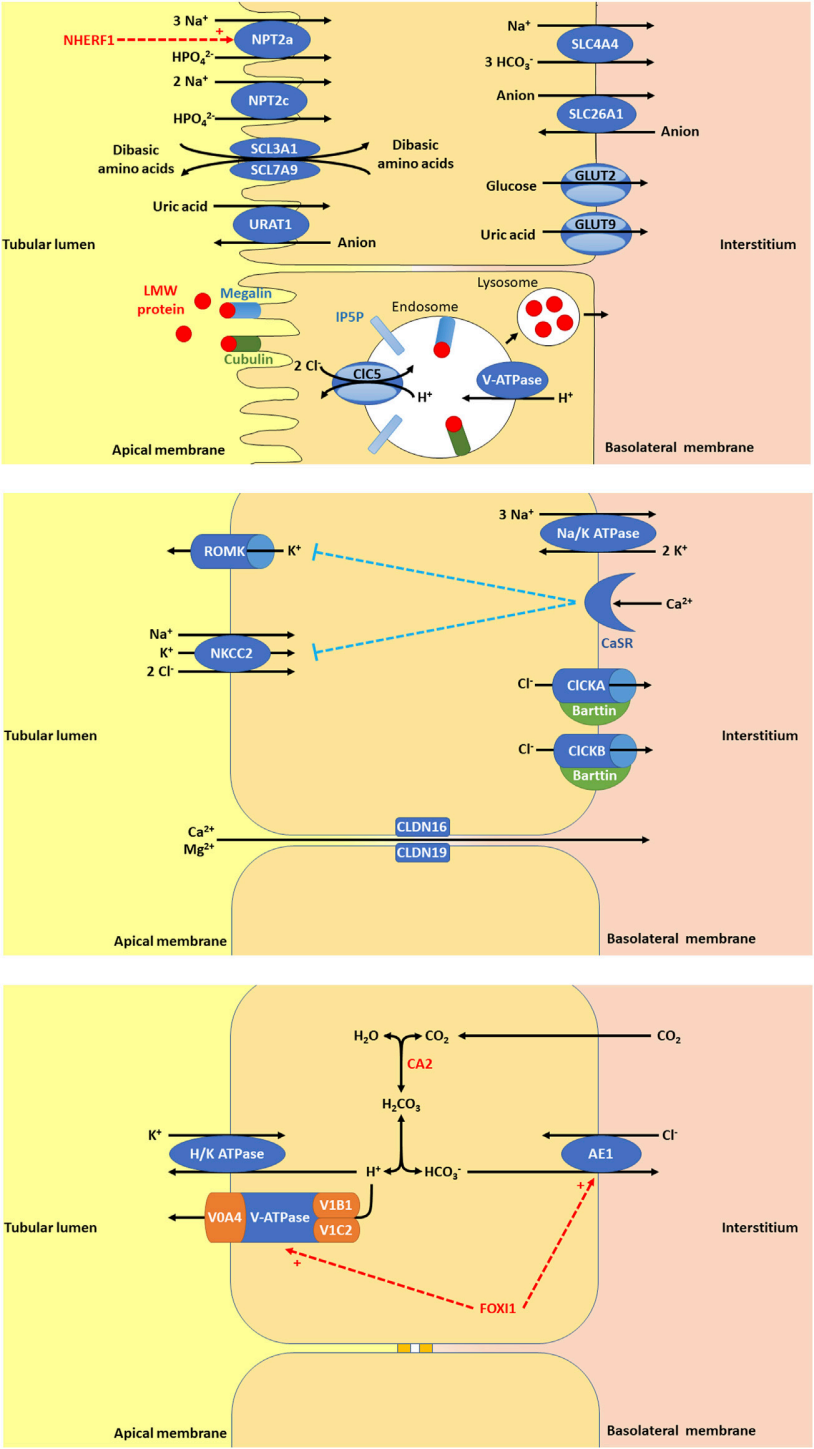
**(A)** In the proximal tubule (PT), there are a variety of apical membrane transporters, basolateral membrane transporters, and endosome transporters proteins that play important roles in PT function. Apical membrane transporters shown are sodium-dependent phosphate transport protein 2a (NPT2a, encoded by *SLC34A1,* whose activity is promoted by NHERF1, sodium/hydrogen exchange regulatory factor 1, encoded by *SLC9A3R1*), sodium-dependent phosphate transport protein 2c (NPT2c, encoded by *SLC34A3*), SLC3A1/SLC7A9 (encoded by *SLC3A1* and *SLC7A9*), and urate-anion transporter (URAT1, encoded by *SLC22A12*). The basolateral membrane transporters shown are glucose transporter 2 (GLUT2, encoded for by *SLC2A2*), facilitated glucose transporter 9 (GLUT9)/voltage-driven urate transporter (encoded for by *SLC2A9*), solute carrier family 4 member 4 (SLC4A4, a sodium bicarbonate transporter encoded by *SLC4A4*), and solute carrier family 26 member 1 (SLC26A1, an electroneutral anion exchanger encoded by *SLC26A1*). Low molecular weight (LMW) protein in the tubular lumen binds megalin and cubulin and are endocytosed into endosomes, thought to be facilitated by inositol polyphosphate-5-phosphatase (IP5P), ended by *OCRL*, which is expressed in the glomerulus, the PT, the thick ascending loop of Henle (TAL), the distal convoluted tubule (DCT) and the collecting duct (CD). Endosome acidification is then mediated by V-ATPase and the chloride voltage-gated channel 5 (ClC5, encoded by *CLCN5,* expressed in the PT, alpha-intercalated cell (alpha-IC) and the TAL. Lysosomes form to allow the absorption of LMW protein and recycling of megalin and cubulin to the apical membrane. **(B)** In the TAL, there are a variety of apical membrane transporters, tight junction proteins, and basolateral membrane transporters, receptors, and proteins that play important roles in tubular function. Apical membrane transporters shown are kidney-specific Na-K-Cl symporter (NKCC2) and renal out-medullar potassium channel (ROMK). NKCC2 (encoded by *SLC12A1*) that is responsible for sodium, potassium, and chloride reabsorption from the tubular lumen. ROMK (encoded by *KCNJ1*) is responsible for excretion of potassium. The tight junction proteins shown are claudin 16 (CLDN16, encoded by *CLDN16*) and claudin 19 (CLDN19, encoded by *CLDN19*). CLDN16 and CLDN19 are necessary for paracellular reabsorption of calcium and magnesium. The basolateral membrane transporters and receptors shown include Na/K ATPase, chloride voltage-gated channel kidney A (ClCKA), chloride voltage-gated channel kidney B (ClCKB), Barttin, and calcium sensing receptor (CaSR). Na/K ATPase is responsible for generating the gradient for sodium entry across apical membranes via NKCC2 and subsequent exit of chloride across the basolateral membrane via ClCKA, ClCKB, and Barttin. ClCKA is encoded for by *CLCNKA* and ClCKB is encoded for by *CLCNKB.* These chloride channels allow chloride to exit the cell via the basolateral membrane. Barttin or chloride voltage-gated channel kidney (ClCK)-type accessory subunit beta, encoded for by *BSND.* ClCKA and ClCKB channels depend on the presence of the Barttin subunit for chloride transport. CaSR (encoded for by *CASR*), is expressed in the parathyroid gland and the kidney in the TAL, the PT, the DCT, and the CD. In the TAL, activation of CaSR by calcium inhibits NaCl reabsorption by inhibiting NKCC2 and ROMK. By inhibiting ROMK, the tubular lumen positive voltage is diminished, reducing the driving force for paracellular cation (including calcium) reabsorption. **(C)** In the alpha-IC of the CD, there are few key, apical membrane transporters, and basolateral membrane transporters that play important roles in tubular function. Carbonic anhydrase 2 (CA2) is an enzyme encoded by *CA2* that is expressed in the CD (including the alpha-IC) and PT. CA2 catalyzes the combination of carbon dioxide and water to form carbonic acid (H_2_CO_3_), which then dissociates to protons (H^+^) and bicarbonate (HCO_3_^−^). The protons produced are excreted into the urine by exiting the cell through the apical membrane via H/K ATPase and V-ATPase, a proton transporter with V0 subunit A4 (V0A4, encoded by *ATP6V0A4*), V0 subunit B1 (V1B1, encoded by *ATP6V1A1*), and V1 subunit C2 (V1C2, encoded by *ATP6V1C2*). The bicarbonate produced is reabsorbed into the interstitium by exiting the cell thought the basolateral membrane via anion exchange protein 1 (AE1), a basolateral chloride bicarbonate counter transporter encoded by *SLC4A1.* Forkhead box I1 (FOXI1) encoded for by *FOXI1* is a transcription factor that regulates the function of AE1 and V-ATPase.

*CASR* encodes for the calcium-sensing receptor (CaSR), which is important in renal calcium homeostasis and expressed in the parathyroid gland and the kidney in the thick ascending loop of Henle (TAL) basolateral membrane (Figure 1), the PT, the distal convoluted tubule (DCT), and the CD (Brown and MacLeod, 2001). In the TAL, activation of CaSR by calcium inhibits NaCl reabsorption by inhibiting NKCC2 and ROMK (Gamba and Friedman, 2009). By inhibiting ROMK, the tubular lumen positive voltage is diminished, reducing the driving force for paracellular cation (including calcium) reabsorption (Gamba and Friedman, 2009).

AD hypocalcemia/AD hypocalcemia with Bartter syndrome (OMIM phenotype number 601198) is a condition resulting from activating variants in *CASR*, which increase the sensitivity of CaSR to extracellular calcium (Brown and MacLeod, 2001). Activation of CaSR in the parathyroid gland leads to inhibition of parathyroid hormone (PTH) release and subsequent hypoparathyroidism (Brown and MacLeod, 2001). Reduced PTH release leads to decreased calcium and phosphorus resorption in the bone (Brown and MacLeod, 2001). In the kidney, reduced PTH leads to decreased TAL and DCT reabsorption of calcium, increased reabsorption of phosphorus, and decreased production of 1,25-dihydroxyvitamin D3, leading to decreased absorption of calcium in the intestine (Brown and MacLeod, 2001). All of the above culminates in hypocalcemia, hypercalciuria, NC, and NL (Pearce et al., 1996). Some may have features of Bartter syndrome such as impaired sodium chloride reabsorption in the TAL, hypokalemic metabolic alkalosis, hyperreninemia and/or hyperaldosteronism (Vargas-Poussou et al., 2002). Treatment with minimal doses of calcium and vitamin D administration may be given to alleviate symptoms, but excess can exacerbate hypercalciuria, NC, and lead to kidney impairment (Pearce et al., 1996).

Neonatal hyperparathyroidism (OMIM phenotype number 239200) is an AD/AR condition resulting from inactivating variants in *CASR* that decrease the sensitivity of CaSR to extracellular calcium (Brown and MacLeod, 2001). Inactivation of CaSR in the parathyroid gland leads to stimulation of PTH release and subsequent hyperparathyroidism (Lila et al., 2012; El Allali et al., 2021). Increased PTH release leads to increased calcium and phosphorus resorption in the bone (Lila et al., 2012; El Allali et al., 2021). In the kidney, increased PTH leads to increased tubular reabsorption of calcium, decreased reabsorption of phosphorus, and increased production of 1,25-dihydroxyvitamin D3, leading to increased absorption of calcium in the intestine (Lila et al., 2012; El Allali et al., 2021). This culminates in severe hypercalcemia, NC, and NL (Lila et al., 2012; El Allali et al., 2021). Calcimimetic medications may be given treat this condition by increasing sensitivity of CaSR to extracellular calcium (El Allali et al., 2021).

Hypocalciuric hypercalcemia type 1 (OMIM phenotype number 145980) is an AD condition resulting from an inactivating variant in *CASR*, resulting in lifelong mild to moderate hypercalcemia, inappropriate hypocalciuria, and normal PTH to mild hyperparathyroidism (Carling et al., 2000). This condition may also have atypical presentations with severe hypercalcemia and hypercalciuria with or without NL or NC (Carling et al., 2000).

##### 3.1.1.2 CYP24A1 gene

*CYP24A1* encodes for cytochrome P450 family 24 subfamily A member 1 or 1,25-dihydroxyvitamin D3 24-hydroxylase, the enzyme primarily responsible for the catabolism of 1,25-dihydroxy- and 25-hydroxy-vitamin D, which is expressed in the PT (Schlingmann et al., 2011). Infantile hypercalcemia 1 (OMIM phenotype number 143880) is an AR condition resulting from an inactivating variant in *CYP24A1*, resulting in accumulation of 1,25-dihydroxyvitamin D3 (Schlingmann et al., 2011). This accumulation leads to severe hypercalcemia, failure to thrive, vomiting, dehydration, NC, and NL (Schlingmann et al., 2011). Data suggests that carriers (heterozygotes) of inactivating variants in *CYP24A1* may have increased risk of NC and NL (Carpenter, 2017).

For treatment of Infantile hypercalcemia 1, generous hydration and a diet low in vitamin D and calcium are suggested (Schlingmann et al., 2011). CYP3A4 inducers (rifampin) or CYP27B1 modulators (fluconazole/ketoconazole/itraconazole) may help reduce 1,25-dihydroxyvitamin D3 levels and improve hypercalcemia and hypercalciuria (Sayers et al., 2015; Hawkes et al., 2017). Low doses of these medications are suggested, but the long-term safety is uncertain.

#### 3.1.2 Renal phosphate wasting with hypercalciuria

Genetic causes of renal phosphate wasting with hypercalciuria and NL and/or NC are due to variants in *SCL34A1*, *SLC34A3*, and *SLC9A3R1* (Table 2). These individuals typically require treatment with phosphate supplementation (Tieder et al., 1992).

**TABLE 2:** Genetic causes of renal phosphate wasting with hypercalciuria and nephrolithiasis and/or nephrocalcinosis.

Gene	Gene product	Phenotype	OMIM phenotype number	Inheritance	Description
*SLC34A1*	Solute carrier family 34 member 1, sodium-dependent phosphate transport protein 2a (NPT2a) (PT)	Infantile hypercalcemia 2 (Schlingmann et al., 2016)	616963	AR	Decreased renal phosphate absorption, hypophosphatemia, high 1,25-dihydroxyvitamin D3, hypercalcemia, hypercalciuria, failure to thrive, vomiting, dehydration, NC
		Hypophosphatemic NL/osteoporosis 1 (Prié et al., 2002)	612286	AD	Decreased renal phosphate absorption, hypophosphatemia, high 1,25-dihydroxyvitamin D3, hypercalciuria, osteoporosis, recurrent NL
*SLC34A3*	Solute carrier family 34 member 3, sodium-dependent phosphate transport protein 2c (NPT2c) (PT)	Hypophosphatemic rickets with hypercalciuria (Bergwitz et al., 2006)	241530	AR	Decreased renal phosphate absorption, hypophosphatemia, increased serum 1,25-dihydroxyvitamin D levels, hypercalciuria, rickets, muscle weakness, bone pain, NL, NC
		SLC34A3 carrier (Dasgupta et al., 2014)	N/A	Carrier	Increased risk for NL, NC, hypercalciuria, hypophosphatemia
*SLC9A3R1*	Solute carrier family 9 member 3 regulator 1, sodium/hydrogen exchange regulatory factor 1 (NHERF1) (PT)	Hypophosphatemic NL/osteoporosis 2 (Prié et al., 2002; Karim et al., 2008; Courbebaisse et al., 2012)	612287	AD	Decreased renal phosphate absorption, hypophosphatemia, high 1,25-dihydroxyvitamin D3, hypercalciuria, osteoporosis, recurrent NL

AD, autosomal dominant; AR, autosomal recessive; NC, nephrocalcinosis; NL, nephrolithiasis; P, proximal tubule.

##### 3.1.2.1 SLC34A1 gene

*SLC34A1* encodes for the solute carrier family 34 member 1, the sodium-dependent phosphate transport protein 2a (NPT2a), which responsible for the majority of renal phosphate transport and is primarily expressed in the PT (Figure 1) (Schlingmann et al., 2016). Fanconi renotubular syndrome 2 (OMIM phenotype number 613388) is one of the conditions caused by homozygous variants in *SLC34A1* but will not be discussed in this review as this condition has not been associated with NL or NC.

Infantile hypercalcemia 2 (OMIM phenotype number 616963) is an AR condition due to inactivating variants in *SLC34A1*, resulting in loss of NPT2a activity and subsequent renal phosphorus wasting with hypophosphatemia (Schlingmann et al., 2016). This hypophosphatemia results in increased production of 1,25-dihydroxyvitmain D3 with hypercalcemia, hypercalciuria, failure to thrive, vomiting, dehydration, and NC (Schlingmann et al., 2016). In addition to phosphate supplementation, generous hydration and a diet low in vitamin D and calcium are also suggested (Schlingmann et al., 2016). CYP3A4 inducers (rifampin) or CYP27B1 modulators (fluconazole/ketoconazole/itraconazole) may help reduce 1,25-dihydroxyvitamin D3 levels and improve hypercalcemia and hypercalciuria (Sayers et al., 2015; Hawkes et al., 2017). Low doses of these medications are suggested, but the long-term safety is uncertain.

Hypophosphatemic NL/osteoporosis 1 (OMIM phenotype number 612286) is an AD condition due to an inactivating variant in SLC34A1, resulting in loss of NPT2a activity and subsequent renal phosphorus wasting with hypophosphatemia (Prié et al., 2002). This hypophosphatemia results in increased production of 1,25-dihydroxyvitmain D3 with hypercalciuria, osteoporosis, and recurrent NL (Prié et al., 2002).

##### 3.1.2.2 SLC34A3 gene

*SLC34A3* encodes for the solute carrier family 34 member 3, the sodium-dependent phosphate transport protein 2c (NPT2c) which is primarily expressed in the PT (Figure 1) (Bergwitz et al., 2006). Hypophosphatemic rickets with hypercalciuria (OMIM phenotype number 241530) is an AR condition due to inactivating variants in *SLC34A3*, resulting in loss of NPT2c activity and subsequent renal phosphorus wasting with hypophosphatemia (Bergwitz et al., 2006). This hypophosphatemia results in increased production of 1,25-dihydroxyvitmain D3 with hypercalcemia, hypercalciuria, rickets, muscle weakness, bone pain, NL, and NC (Bergwitz et al., 2006). Data suggests that carriers (heterozygotes) of inactivating variants in *SLC34A3* may have increased risk of NL, NC, hypercalciuria, and hypophosphatemia (Dasgupta et al., 2014).

##### 3.1.2.3 SLC9A3R1 gene

*SLC9A3R1* encodes for the solute carrier family 9 member 3 regulator 1/sodium/hydrogen exchange regulatory factor 1 (NHERF1), which is primarily expressed in the PT (Figure 1) (Karim et al., 2008; Courbebaisse et al., 2012). Hypophosphatemic NL/osteoporosis 2 (OMIM phenotype number 612287) is an AD condition due to an inactivating variant in *SLC9A3R1*, resulting in loss of NHERF1 activity with subsequent decrease in NPT2a expression, resulting in renal phosphorus wasting with hypophosphatemia (Courbebaisse et al., 2012). This subsequently results in increased production of 1,25-dihydroxyvitmain D3 with hypercalciuria, osteoporosis, and recurrent NL (Prié et al., 2002).

#### 3.1.3 Renal magnesium wasting with hypercalciuria

Genetic causes of renal magnesium wasting with hypercalciuria and NL and/or NC are related to, *CLDN16*, *CLDN19*, and *RRAGD*, and are shown in Table 3. The conditions related to variants in these genes are discussed further below.

**TABLE 3:** Genetic causes of renal magnesium wasting with hypercalciuria and nephrolithiasis and/or nephrocalcinosis.

Gene	Gene product	Phenotype	OMIM phenotype number	Inheritance	Description
*CLDN16*	Claudin 16/paracellin-1, integral membrane tight junction protein (TAL, DCT)	Renal hypomagnesemia 3 (Weber et al., 2001; Godron et al., 2012)	248250	AR	Hypomagnesemia, high urinary magnesium excretion, hypercalciuria, NC, NL, progressive CKD
		CLDN16 carrier (Deeb et al., 2013)	N/A	Carrier	May have hypercalciuria, NL
*CLDN19*	Claudin 19, integral membrane tight junction protein (TAL, DCT)	Renal hypomagnesemia 5 with ocular involvement (Weber et al., 2001; Godron et al., 2012)	248190	AR	Visual impairment, hypomagnesemia, high urinary magnesium excretion, hypercalciuria, NC, NL, progressive CKD
		CLDN19 carrier (Naeem et al., 2011)	N/A	Carrier	May have NL
*RRAGD*	Ras related GTP binding D (TAL, DCT)	Renal hypomagnesemia 7 with or without dilated cardiomyopathy (Schlingmann et al., 2021)	620152	AD	Renal salt wasting resulting in hypomagnesemia, polyuria, hypokalemia, hypochloremia, metabolic alkalosis, hypercalciuria, many develop NC, not typically associated with CKD, some develop severe dilated cardiomyopathy

AD, autosomal dominant; AR, autosomal recessive; CD, collecting duct; CKD, chronic kidney disease; DCT, distal convoluted tubule; NC, nephrocalcinosis; NL, nephrolithiasis; TAL, thick ascending loop of Henle.

##### 3.1.3.1 CLDN16 gene

*CLDN16* encodes for Claudin 16 or paracellin-1, which is an integral membrane tight junction protein necessary for paracellular reabsorption of calcium and magnesium (Godron et al., 2012). *CLDN16* is expressed in the TAL and DCT (Figure 1). Renal hypomagnesemia 3 (OMIM phenotype number 248250) is an AR condition due to inactivating variants in *CLDN16*, resulting reduced reabsorption of calcium (hypercalciuria)and magnesium (hypermagnesuria) with hypomagnesemia, NC, NL, and progressive CKD (Godron et al., 2012). Data suggests that carriers (heterozygotes) of inactivating variants in *CLDN16* may have hypercalciuria and NL (Deeb et al., 2013).

Medical management of Renal hypomagnesemia 3 typically involves high-dose magnesium supplementation to replace renal losses, although this has not been shown to slow progression of CKD (Weber et al., 2001; Godron et al., 2012). Thiazide diuretics and indomethacin have been used in this condition but have not been shown to be successful in reducing hypercalciuria or hypermagnesuria or preventing progression of CKD (Weber et al., 2001; Godron et al., 2012). Definitive treatment can be achieved with kidney transplantation (Weber et al., 2001).

##### 3.1.3.2 CLDN19 gene

*CLDN19* encodes for Claudin 19, which is an integral membrane tight junction protein necessary for paracellular reabsorption of calcium and magnesium (Godron et al., 2012). *CLDN19* is expressed in the TAL and DCT (Figure 1). Renal hypomagnesemia 5 with ocular involvement (OMIM phenotype number 248190) is an AR condition due to inactivating variants in *CLDN19*, resulting reduced reabsorption of calcium (hypercalciuria) and magnesium (hypermagnesuria)with hypomagnesemia, visual impairment, NC, NL, and progressive CKD (Godron et al., 2012). Data suggests that carriers (heterozygotes) of inactivating variants in *CLDN16* may have NL (Naeem et al., 2011). Medical management of Renal hypomagnesemia 5 with ocular involvement and its efficacy are typically the same as that for Renal hypomagnesemia 3 caused by variants in *CLDN16* (Weber et al., 2001; Godron et al., 2012).

##### 3.1.3.3 RRAGD gene

*RRAGD* encodes for Ras related GTP binding D, which is expressed in the TAL and DCT (Schlingmann et al., 2021). Renal hypomagnesemia 7 with or without dilated cardiomyopathy (OMIM phenotype number 620152) is an AD condition due to an inactivating variant in *RRAGD*, resulting in activation of mTOR signaling, which leads to renal salt wasting resulting in hypomagnesemia, polyuria, hypokalemia, hypochloremia, metabolic alkalosis, hypercalciuria, and NC (Schlingmann et al., 2021). This condition is not typically associated with CKD, and some individuals develop severe dilated cardiomyopathy (Schlingmann et al., 2021). Optimal treatment strategy is unclear.

#### 3.1.4 Distal renal tubular acidosis

Distal RTA results from the impaired ability to acidify the urine, which in turn leads to hyperchloremic metabolic acidosis (Karet, 2002). Distal RTA is associated and hypocitraturia with alkaline urine due to upregulation of citrate reabsorption in the PT, hypercalciuria and resultant NC and/or NL, and may be associated with hypokalemia (Karet, 2002; Lopez-Garcia et al., 2019). Treatment for distal RTA primarily consists of alkali therapy to correct metabolic acidosis, with potassium-containing medications such as potassium citrate being the preferred choice (Mohebbi and Wagner, 2018). Thiazide diuretics may be considered with severe hypercalciuria but may be complicated by polyuria and hypokalemia (Mohebbi and Wagner, 2018). Many individuals with chronic untreated or severe metabolic acidosis develop rickets or osteomalacia, although it appears as though most adults achieve normal adult height, especially those with better control of acidosis (Karet, 2002; Lopez-Garcia et al., 2019). CKD stages 2–4 have been reported in >80% of adults and in >30% of children with distal RTA with ESKD rarely occurring (Lopez-Garcia et al., 2019). In individuals with better control of acidosis, CKD incidence is lower (Lopez-Garcia et al., 2019). Table 4 shows genetic causes of distal RTA with hypercalciuria and NL and/or NC.

**TABLE 4:** Genetic causes of distal renal tubular acidosis with hypercalciuria and nephrolithiasis and/or nephrocalcinosis.

Gene	Gene product	Phenotype	OMIM phenotype number	Inheritance	Description
*ATP6V0A4*	V-ATPase H+ transporting V0 subunit A4 (alpha-IC)	Distal RTA type 3 with or without sensorineural hearing loss (Karet, 2002; Lopez-Garcia et al., 2019)	602722	AR	Distal RTA usually accompanied by NC and/or NL, may have later-onset sensorineural deafness
		ATP6V0A4 carrier (Imai et al., 2016)	N/A	Carrier	May have incomplete distal RTA associated with NC
*ATP6V1B1*	V-ATPase H+ transporting V1 subunit B1 (alpha-IC)	Distal RTA type 2 with progressive sensorineural hearing loss (Karet, 2002; Lopez-Garcia et al., 2019)	267300	AR	Early-onset sensorineural deafness, distal RTA usually accompanied by NC and/or NL
		ATP6V1B1 carrier (Dhayat et al., 2016)	N/A	Carrier	May have incomplete distal RTA, associated with recurrent NL.
*ATP6V1C2*	V-ATPase H+ transporting V1 subunit C2 (alpha-IC)	Distal RTA (Jobst-Schwan et al., 2020)	N/A	AR	Distal RTA, likely associated with NC and/or NL, early ESKD
*FOXI1*	Forkhead box I1 (alpha-IC)	Distal RTA and early-onset deafness (Enerbäck et al., 2018)	N/A	AR	Early-onset sensorineural deafness, distal RTA, NC
*SLC4A1*	Basolateral chloride bicarbonate counter transporter anion exchange protein 1 (AE1) (alpha-IC)	Distal RTA 1 (Karet, 2002; Lopez-Garcia et al., 2019)	179800	AD	Associated with NC and/or NL
		Distal RTA 4 with hemolytic anemia (Sritippayawan et al., 2004; Lopez-Garcia et al., 2019)	611590	AR	Associated with hemolytic anemia, NC

AD, autosomal dominant; Alpha-IC, alpha-intercalated cell; AR, autosomal recessive; ESKD, end stage kidney disease; NC, nephrocalcinosis; NL, nephrolithiasis; RTA, renal tubular acidosis.

##### 3.1.4.1 ATP6V0A4 gene

*ATP6V0A4* encodes for V-ATPase H+ transporting V0 subunit A4, which excretes protons into the urine and is expressed in the alpha-IC of the CD (Figure 1) (Karet, 2002). Distal RTA type 3 with or without sensorineural hearing loss (OMIM phenotype number 602722) is an AR condition due to inactivating variants in *ATP6V0A4*, resulting in decreased urinary proton excretion, which leads to a distal RTA usually accompanied by NC and/or NL as well later-onset sensorineural deafness in >30% (Karet, 2002; Lopez-Garcia et al., 2019). Data suggests that carriers (heterozygotes) of inactivating variants in *ATP6V0A4* may have an incomplete distal RTA associated with NC (Imai et al., 2016).

##### 3.1.4.2 ATP6V1B1 gene

*ATP6V1B1* encodes for V-ATPase H+ transporting V1 subunit B1, which excretes protons into the urine and is expressed in the alpha-IC of the CD (Figure 1) (Karet, 2002). Distal RTA type 2 with progressive sensorineural hearing loss (OMIM phenotype number 267300) is an AR condition due to inactivating variants in *ATP6V1B1*, resulting in decreased urinary proton excretion, which leads to a distal RTA usually accompanied by NC and/or NL as well early-onset sensorineural deafness in most (Lopez-Garcia et al., 2019). Data suggests that carriers (heterozygotes) of inactivating variants in *ATP6VAB1* may have an incomplete distal RTA associated with recurrent NL (Dhayat et al., 2016).

##### 3.1.4.3 ATP6V1C2 gene

*ATP6V1C2* encodes for V-ATPase H+ transporting V1 subunit C2, which excretes protons into the urine and is expressed in the alpha-IC of the CD (Figure 1) (Jobst-Schwan et al., 2020). *ATP6V1C2*-associated distal RTA is an AR condition due to inactivating variants in *ATP6V1C2*, resulting in decreased urinary proton excretion, which leads to a distal RTA that is likely associated with NC and/or NL and with early ESKD (Jobst-Schwan et al., 2020).

##### 3.1.4.4 SLC4A1 gene

*SLC4A1* encodes for the basolateral chloride bicarbonate counter transporter anion exchange protein 1 (AE1), which reabsorbs bicarbonate from the urine into the circulation and is expressed in the alpha-IC of the CD (Figure 1) (Karet, 2002). Distal RTA 1 (OMIM phenotype number 179800) is an AD condition due to an inactivating variant in *SLC4A1*, resulting in decreased urinary bicarbonate reabsorption, which leads to a distal RTA usually accompanied by NC and NL (Karet, 2002; Lopez-Garcia et al., 2019). Distal RTA 4 with hemolytic anemia (OMIM phenotype number 611590) is an AR condition due to inactivating variants in *SLC4A1*, resulting in decreased urinary bicarbonate reabsorption, which leads to a distal RTA with hemolytic anemia and NC. (Sritippayawan et al., 2004; Lopez-Garcia et al., 2019).

##### 3.1.4.5 FOXI1 gene

*FOXI1* encodes for Forkhead box I1, which is a transcription factor that regulates the function of AE1, AE4, and V-ATPase subunits, and is expressed in the alpha-IC of the CD (Figure 1) (Enerbäck et al., 2018). Distal RTA and early-onset deafness is an AR condition due to inactivating variants in *FOXI1*, resulting in decreased urinary bicarbonate reabsorption, which leads to a distal RTA with early-onset sensorineural deafness and NC (Enerbäck et al., 2018).

#### 3.1.5 Proximal tubulopathy

Proximal tubulopathy consists of dysfunction of the PT, which can lead to any combination of low molecular weight (LMW) proteinuria, aminoaciduria, glucosuria, urine bicarbonate wasting and RTA, hypercalciuria, hyperphosphaturia, high urinary potassium excretion, and uricosuria. NC and NL can occur with proximal tubulopathies, but less commonly so than with distal RTA. Another name for proximal tubulopathy is Fanconi renotubular syndrome. Treatment generally consists of supplementation to treat urinary solute losses.

Supplementary Table S2 contains a list of genetic causes of secondary proximal tubulopathy with hypercalciuria and NL and/or NC. Conditions that results in a secondary proximal tubulopathy include Hereditary fructose intolerance (*ALDOB* gene, AR inheritance (Higgins and Varney, 1966; Mass et al., 1966), OMIM phenotype number 229600), Wilson disease (*ATP7B* gene, AR inheritance, OMIM phenotype number 277900) (Di Stefano et al., 2012), Nephropathic cystinosis (*CTNS* gene, AR inheritance, OMIM phenotype number 219800) (Theodoropoulos et al., 1995), Tyrosinemia type 1 (*FAH* gene, AR inheritance, OMIM phenotype number 276700) (Forget et al., 1999), Congenital lactase deficiency (*LCT* gene, AR inheritance, OMIM phenotype number 223000) (Saarela et al., 1995), Mitochondrial DNA depletion syndrome 8A (encephalomyopathic type with renal tubulopathy) (*RRM2B* gene, AR inheritance, OMIM phenotype number 612075) (Finsterer and Scorza, 2017), Sideroblastic anemia with B-cell immunodeficiency, periodic fevers, and developmental delay (*TRNT1* gene, AR inheritance, OMIM phenotype number 616084) (Wiseman et al., 2013), Arthrogryposis, renal dysfunction, and cholestasis 1 (*VPS33B* gene, AR inheritance, OMIM phenotype number 208085) (Holme et al., 2013), and Arthrogryposis, renal dysfunction, and cholestasis 2 (*VIPAS39* gene, AR inheritance, OMIM phenotype number 613404) (Holme et al., 2013).

Primary inherited proximal tubulopathies associated with NC and/or NL are shown in Table 5 and will be discussed in greater detail below. Inactivating variants in *EHHADH* (encodes for enoyl-CoA hydratase and 3-hydroxyacyl CoA dehydrogenase) cause AD Fanconi renotubular syndrome 3 (OMIM phenotype number 615605), is associated with rickets, impaired growth, glucosuria, generalized aminoaciduria, phosphaturia, metabolic acidosis, LMW proteinuria, and hypercalciuria will not be discussed as there is no known association with NC or NL (Tolaymat et al., 1992; Klootwijk et al., 2014).

**TABLE 5:** Genetic causes of primary proximal tubulopathy with hypercalciuria and nephrolithiasis and/or nephrocalcinosis.

Gene	Gene product	Phenotype	OMIM phenotype number	Inheritance	Description
*CLCN5*	Chloride voltage-gated channel 5 (CLC5), chloride/proton exchanger (PT, alpha-IC, TAL)	Dent disease 1 (Claverie-Martín et al., 2011; Blanchard et al., 2016)	300009	XLR	Progressive proximal tubulopathy, LMW proteinuria, hypercalciuria, NC, NL, progressive ESKD
		NL type 1 (Frymoyer et al., 1991)	310468	XLR	Progressive proximal tubulopathy, LMW proteinuria, hypercalciuria, NC, NL, progressive ESKD
		Hypo-phosphatemic rickets (Bolino et al., 1993)	300554	XLR	Progressive proximal tubulopathy, LMW proteinuria, rickets or osteomalacia, hypophosphatemia, hypercalciuria, NC, NL, progressive ESKD
		LMW proteinuria with hypercalciuric NC (Igarashi et al., 1995)	308990	XLR	Progressive proximal tubulopathy, LMW proteinuria, hypercalciuria, NC, NL, without progressive ESKD
		Female CLCN5 carrier (Hoopes et al., 1998)	N/A	Female carrier	May have mild symptoms of Dent disease (LMW proteinuria, hypercalciuria), few reported cases of NL, progressive CKD
*OCRL*	Inositol polyphosphate-5-phosphatase (glomerulus, PT, TAL, DCT, CD)	Dent disease 2 (Claverie-Martín et al., 2011)	300555	XLR	Progressive proximal tubulopathy, LMW proteinuria, hypercalciuria, NC, NL, progressive ESKD
		Lowe syndrome (Claverie-Martín et al., 2011)	309000	XLR	Hydrophthalmia, cataracts, ID, vitamin D-resistant rickets, progressive proximal tubulopathy, LMW proteinuria, proximal RTA hypercalciuria, NC, NL, progressive ESKD
		Female OCRL carrier (Cau et al., 2006)	N/A	Female carrier	Usually asymptomatic, may have Dent disease 2, Lowe syndrome
*GATM*	Glycine amidino-transferase (PT)	Fanconi renotubular syndrome 1 (Wen et al., 1989; Reichold et al., 2018; Seaby et al., 2023)	134600	AD	Decreased solute and water reabsorption in PT, polydipsia, polyuria, phosphaturia, glycosuria, aminoaciduria, uricosuria, hypophosphatemic rickets, metabolic acidosis, progressive CKD, and at least 1 reported case of NC
*HNF4A*	Hepatocyte nuclear factor 4-alpha (PT)	Fanconi renotubular syndrome 4 with maturity-onset diabetes of the young (Hamilton et al., 2014)	616026	AD	Macrosomia, severe hypoglycemia with hyperinsulinism, rickets, LMW proteinuria, aminoaciduria, glycosuria, phosphaturia, hypercalciuria, hypouricemia, metabolic acidosis, NC
*SLC2A2*	Solute carrier family 2 (facilitated glucose transporter) member 2/glucose transporter 2 (GLUT2) (PT)	Fanconi–Bickel syndrome (Fridman et al., 2015)	227810	AR	Hepatorenal glycogen accumulation, Fanconi renotubular syndrome, impaired utilization of glucose and galactose, hypercalciuria, NC

AD, autosomal dominant; Alpha-IC, alpha-intercalated cell; AR, autosomal recessive; CD, collecting duct; CKD, chronic kidney disease; DCT, distal convoluted tubule; ESKD, end stage kidney disease; ID, intellectual disability; LMW, low molecular weight; NC, nephrocalcinosis; NL, nephrolithiasis; PT, proximal tubule; RTA, renal tubular acidosis; TAL, thick ascending loop of Henle; XLR, X-linked recessive.

##### 3.1.5.1 CLCN5 gene

*CLCN5* encodes for chloride voltage-gated channel 5 (CLC5), a chloride/proton exchanger, and is expressed in the PT (Figure 1), alpha-IC, and TAL (Mansour-Hendili et al., 2015). Inactivating variants in *CLCN5* appear to cause abnormal reabsorption of LMW proteins by disrupting PT vesicle acidification, impacting lysosome function and LMW protein processing (Nielsen et al., 2016). Inactivating variants in *CLCN5* are associated with a spectrum of conditions (Claverie-Martín et al., 2011).

Dent disease 1 (OMIM phenotype number 300009) is an XLR condition resulting from inactivating variants in *CLCN5.* Hallmark features are progressive proximal renal tubulopathy with LMW proteinuria, hypercalciuria, NC, NL, and progressive ESKD (Claverie-Martín et al., 2011). Median age at ESKD onset has been reported as 40 years of age (Blanchard et al., 2016). Treatment is aimed at treating serum electrolyte disturbances (such as treating hypophosphatemia with phosphorus supplementation) and minimizing hypercalciuria, primarily with hydration and administration of thiazide diuretics. However, the administration thiazide diuretics to reduce hypercalciuria is controversial as there is no definite correlation between hypercalciuria and ESKD, and significant side effects have been reported including dehydration and hypokalemia (Blanchard et al., 2008). Kidney transplantation has been successful in these patients without recurrence of NL or NC (Scheinman, 1998). Due to the risk of worsening hypercalciuria with vitamin D supplementation, it should be reserved for patients who require it for treatment of rickets.

NL type 1 (OMIM phenotype number 310468) is an XLR condition resulting from inactivating variants in *CLCN5.* Hypophosphatemic rickets (OMIM phenotype number 300554) is an XLR condition resulting from inactivating variants in *CLCN5.* It is characterized by progressive proximal renal tubulopathy with LMW proteinuria, rickets or osteomalacia, hypophosphatemia, hypercalciuria, NC, NL, and progressive ESKD (Bolino et al., 1993). LMW proteinuria with hypercalciuric NC (OMIM phenotype number 308990) is an XLR condition resulting from inactivating variants in *CLCN5.* The presentation of LMW proteinuria with hypercalciuric NC resembles that of the conditions mentioned above, except that these individuals do not experience progressive ESKD (Igarashi et al., 1995). Although these three conditions have different OMIM phenotype numbers, it is unclear if they are truly separate conditions from Dent disease 1 as the spectrum of presenting symptoms are the same (Frymoyer et al., 1991). Data suggests that female carriers of inactivating variants in *CLCN5* may have mild symptoms of Dent disease such as low-molecular-weight proteinuria, and hypercalciuria with few reported cases of NL and kidney insufficiency (Hoopes et al., 1998).

##### 3.1.5.2 OCRL gene

*OCRL* encodes for inositol polyphosphate-5-phosphatase, which is thought to be important in endocytosis and cellular trafficking and is expressed throughout the body, including in the glomerulus, PT (Figure 1), TAL, DCT, and CD (Claverie-Martín et al., 2011). Inactivating variants in *OCRL* appear to cause abnormal protein trafficking required for PT solute reabsorption (Ooms et al., 2009). Inactivating variants in *OCRL* are associated with a spectrum of conditions (Claverie-Martín et al., 2011).

Dent disease 2 (OMIM phenotype number 300555) is an XLR condition resulting from inactivating variants in *OCRL.* Clinical features are shared with that of Dent disease 1 caused by variants in CLCN5 with progressive proximal renal tubulopathy with LMW proteinuria, hypercalciuria, NC, NL, and progressive ESKD (Claverie-Martín et al., 2011). Lowe syndrome (OMIM phenotype number 309000) is an XLR condition resulting from inactivating variants in *OCRL.* The presentation resembles that of Dent disease 2 but also involves other systemic symptoms of hydrophthalmia, cataracts, severely impaired intellectual development, vitamin D-resistant rickets, and proximal RTA (Claverie-Martín et al., 2011). Data suggests that female carriers of inactivating variants in *ORCL* are usually asymptomatic, but may have either Dent disease 2 or Lowe syndrome (Cau et al., 2006). Treatment of Dent disease 2 and Lowe syndrome is the same as for Dent disease 1, including treatment of electrolyte derangements such as acidosis.

##### 3.1.5.3 GATM gene

*GATM* encodes for glycine amidinotransferase, which is expressed in the PT mitochondria (Reichold et al., 2018). An inactivating variant in *GATM* leads to Fanconi renotubular syndrome 1 (OMIM phenotype number 134600), an AD condition associated with decreased solute and water reabsorption in the PT with polydipsia, polyuria, phosphaturia, glycosuria, aminoaciduria, uricosuria, hypophosphatemic rickets, metabolic acidosis, progressive kidney insufficiency, and NC in 1 definitive case, and at least 1 case of AD Fanconi syndrome suspected to be secondary a variant in *GATM*, although genetic testing was not performed (Wen et al., 1989; Reichold et al., 2018; Seaby et al., 2023).

##### 3.1.5.4 HNF4A gene

*HNF4A* encodes for hepatocyte nuclear factor 4-alpha, which in the kidney is expressed in the PT. An inactivating variant in *HNF4A* leads to Fanconi renotubular syndrome 4 with maturity-onset diabetes of the young (OMIM phenotype number 616026), an AD condition associated with macrosomia, severe hypoglycemia with hyperinsulinism, rickets, LMW proteinuria, aminoaciduria, glycosuria, phosphaturia, hypercalciuria, hypouricemia, metabolic acidosis, and NC (Hamilton et al., 2014).

##### 3.1.5.5 SLC2A2 gene

*SLC2A2* encodes for solute carrier family 2 (facilitated glucose transporter) member 2 or glucose transporter 2 (GLUT2), which mediates monosaccharide bidirectional transport in the liver, pancreas, intestines, and the renal PT (Figure 1) (Fridman et al., 2015). Inactivating variants in *SLC2A2* lead to Fanconi–Bickel syndrome (OMIM phenotype number 227810), an AR condition associated with Fanconi renotubular syndrome, hepatorenal glycogen accumulation, impaired utilization of glucose and galactose, hypercalciuria, and NC (Fridman et al., 2015).

#### 3.1.6 Mixed or variable tubulopathy

Genetic causes of mixed or variable tubulopathy with hypercalciuria and NL and/or NC are shown in Table 6. One such condition, Kearns-Sayre syndrome, is associated with various mitochondrial DNA (mtDNA) deletions and a variety of tubulopathies (AR inheritance, OMIM phenotype number 530000) (Finsterer and Scorza, 2017). Treatment for distal RTA primarily consists of alkali therapy to correct metabolic acidosis, and treatment of proximal tubulopathy primarily consists of replacement or urinary solute losses (Mohebbi and Wagner, 2018).

**TABLE 6:** Genetic causes of mixed or variable tubulopathy with hypercalciuria and nephrolithiasis and/or nephrocalcinosis.

Gene	Gene product	Phenotype	OMIM phenotype number	Inheritance	Description
*CA2*	Carbonic anhydrase 2 (CD including alpha-IC, PT)	AR osteopetrosis with RTA 3 (Borthwick et al., 2003)	259730	AR	Osteopetrosis with mild proximal RTA, prominent distal RTA, NC
mtDNA	N/A	Kearns-Sayre syndrome (Finsterer and Scorza, 2017)	530000	Unknown	Various mtDNA deletions and rarely point mutation can be identified, associated with pigmentary retinopathy, chronic progressive external ophthalmoplegia, cardiac conduction abnormality, kidney abnormalities including ESKD, RTA, Bartter-like syndrome with Toni-Debré-Fanconi syndrome, NC
*SLC4A4*	Solute carrier family 4 member 4, (sodium bicarbonate cotransporter) (PT)	Proximal RTA with ocular abnormalities (Guo et al., 2023)	604278	AR	Proximal RTA with ocular abnormalities, impaired intellectual development, rarely NL and NC
		Distal RTA, autoimmune thyroiditis, tooth agenesis, enamel hypomaturation, and pulp stones (Kantaputra et al., 2022)	N/A	AR	Only 1 case of this disease exists, which was associated with NC and NL
*WDR72*	WD repeat domain 72 (alpha-IC, PT)	Amelogenesis imperfecta type IIA3 (Khandelwal et al., 2021)	613211	AR	Associated with amelogenesis imperfecta, distal RTA with intermittent proximal tubulopathy in the setting of acidosis, NC, rarely NL

AR, autosomal recessive; CD, collecting duct; ESKD, end stage kidney disease; mtDNA, mitochondrial DNA; NC, nephrocalcinosis; NL, nephrolithiasis; PT, proximal tubule; RTA, renal tubular acidosis.

##### 3.1.6.1 CA2 gene

*CA2* encodes for carbonic anhydrase 2 (CA2), which is expressed in the CD (including the alpha-IC) and PT (Figure 1). CA2 catalyzes the combination of carbon dioxide and water to form carbonic acid, which then dissociates to protons and bicarbonate (Whyte, 1993). Inactivating variants in *CA2* lead to decreased proton and bicarbonate production in osteoclasts and the renal tubules and is associated AR Osteopetrosis with RTA 3 (OMIM phenotype number 259730). This condition is associated with both a proximal RTA and a distal RTA (Borthwick et al., 2003).

##### 3.1.6.2 SLC4A4 gene

*SLC4A4* encodes for solute carrier family 4 member 4, a sodium bicarbonate cotransporter that is important in the reabsorption of bicarbonate in the PT (Figure 1) (Guo et al., 2023). Inactivating variants in this gene are associated with two different conditions. Proximal RTA with ocular abnormalities (OMIM phenotype number 604278) is an AR condition associated with impaired intellectual development, and rarely NL and NC (Guo et al., 2023). Distal RTA, autoimmune thyroiditis, tooth agenesis, enamel hypomaturation, and pulp stones is an AR condition with only 1 case reported with the only case being associated with NC and NL (Kantaputra et al., 2022).

##### 3.1.6.3 WDR72 gene

*WDR72* encodes for WD repeat domain 72, implicated in the trafficking of V-ATPases and thought to play a role in sustained intracellular CaSR signaling through clathrin-mediated endocytosis (Khandelwal et al., 2021). *WDR72* is expressed in the alpha-IC in the CD and PT (Khandelwal et al., 2021). Inactivating variants lead to the AR condition Amelogenesis imperfecta type IIA3 (OMIM phenotype number 613211) associated with a distal RTA and intermittent proximal tubulopathy in the setting of acidosis (Khandelwal et al., 2021). This condition is associated with NC and rarely NL.

#### 3.1.7 Bartter syndrome

Bartter syndrome consists of a group of channelopathies that affect transporter proteins primarily in the TAL involved in sodium chloride reabsorption, which lead to urinary sodium losses, metabolic alkalosis, hypokalemia, hyperaldosteronism and/or hyperreninemia, and hypercalciuria due to loss of paracellular calcium reabsorption (Besouw et al., 2020). There are multiple forms of Bartter syndrome, all of which have been associated with NC (Table 7).

**TABLE 7:** Genetic causes of Bartter syndrome with hypercalciuria and nephrolithiasis and/or nephrocalcinosis.

Gene	Gene product	Phenotype	OMIM phenotype number	Inheritance	Description
*BSND*	Barttin, chloride voltage-gated channel kidney (ClCK)-type accessory subunit beta (TAL, DCT, CD)	Bartter syndrome type 4a (Besouw et al., 2020)	602522	AR	Antenatal polyhydramnios, impaired TAL NaCl reabsorption, hypokalemic metabolic alkalosis, high renin/aldosterone, SNHL, sometimes hypercalciuria, NC
*CLCNKB*	Chloride voltage-gated channel kidney B (ClCKB) (TAL, DCT, CD)	Bartter syndrome 3 (Besouw et al., 2020)	607364	AR	Childhood presentation, impaired TAL NaCl reabsorption, hypokalemic metabolic alkalosis, high renin/aldosterone, sometimes hypercalciuria, NC
*CLCNKA* &*CLCNKB*	Chloride voltage-gated channel kidney A (ClCKA) (tAL, TAL) and Chloride voltage-gated channel kidney B (ClCKB) (TAL, DCT, CD)	Bartter syndrome type 4b (Besouw et al., 2020)	613090	DR	Antenatal polyhydramnios, impaired TAL NaCl reabsorption, hypokalemic metabolic alkalosis, high renin/aldosterone, SNHL, sometimes hypercalciuria, NC
*KCNJ1*	Potassium inwardly rectifier channel subfamily J member 1 or Renal outer-medullary potassium channel (ROMK) (TAL, DCT, CD)	Bartter syndrome 2 (Besouw et al., 2020)	241200	AR	Antenatal polyhydramnios, prematurity, postnatal transient hyperkalemia, impaired TAL NaCl reabsorption, high renin/aldosterone, hypercalciuria, NC
*MAGED2*	Melanoma-associated antigen family member D2 (TAL)	Transient antenatal Bartter syndrome type 5 (Besouw et al., 2020)	300971	XLR	Antenatal polyhydramnios, prematurity, postnatal polyuria with impaired TAL NaCl reabsorption, hyponatremia, hypokalemia, high renin/aldosterone, hypercalciuria, NC, resolves in 1 week
*SLC12A1*	Solute carrier family 12 member 1/kidney-specific Na-K-Cl symporter (NKCC2) (TAL)	Bartter syndrome type 1 (Besouw et al., 2020)	601678	AR	Antenatal polyhydramnios, prematurity, impaired TAL NaCl reabsorption, hypokalemic metabolic alkalosis, high renin/aldosterone, hypercalciuria, NC

AR, autosomal recessive; CD, collecting duct; DCT, distal convoluted tubule; NaCl, sodium chrloride; NC, nephrocalcinosis; NL, nephrolithiasis; SNHL, sensorineural hearing loss; TAL, thick ascending loop of Henle; tAL, thin ascending loop of Henle; XLR, X-linked recessive.

Antenatal in presentation is present for Bartter syndrome type 1 (*SLC12A1* gene, encodes for kidney-specific Na-K-Cl symporter [NKCC2], AR inheritance, OMIM phenotype number 601678) (Figure 1), Bartter syndrome type 2 (*KCNJ1* gene, encodes for renal out-medullar potassium channel [ROMK], AR inheritance, OMIM phenotype number 241200) (Figure 1), Bartter syndrome type 4a (*BSND* gene, encodes for Barttin, AR inheritance, OMIM phenotype number 602522) (Figure 1), Bartter syndrome type 4b (*CLCKA* and *CLCKB* genes, encode for chloride voltage-gated channel kidney A [ClCKA] and chloride voltage-gated channel kidney B [ClCKB], digenic recessive [DR] inheritance, OMIM phenotype number 613090) (Figure 1), and Bartter syndrome type 5 (*MAGED2* gene, XLR inheritance, OMIM phenotype number 300971) (Besouw et al., 2020). However, Bartter syndrome type 5 is transient and resolves after 1 week (Besouw et al., 2020). The only Bartter syndrome that presents in childhood is Bartter syndrome type 3 (*CLCKB* gene, encode for ClCKB, AR inheritance, OMIM phenotype number 607364) (Figure 1) (Besouw et al., 2020). Mainstays of treatment are hydration, sodium and potassium repletion, and nonsteroidal anti-inflammatory drugs (NSAIDs) that help to minimize urinary losses of electrolytes and water (Besouw et al., 2020).

#### 3.1.8 Hyperaldosteronism and pseudohyperaldosteronism

Genetic causes of hyperaldosteronism and pseudohyperaldosteronism with hypercalciuria and NL and/or NC are shown in Supplementary Table S3. Studies have shown that individuals with hyperaldosteronism have a higher incidence of NL compared to the general population (Chang et al., 2022). Hyperaldosteronism has also been associated with NC, thought to be related to factors including increased sodium excretion and resultant hypercalciuria, hyperphosphaturia, hypocitraturia, and hypokalemia-associated ammonia-medicated nephropathy (Mittal et al., 2015). Genetic hyperaldosteronism conditions associated with NL and/or NC are Familial hyperaldosteronism type I/Glucocorticoid-remediable aldosteronism (*CYP11B1* gene, AD inheritance, OMIM phenotype number 103900), Familial hyperaldosteronism type II (*CLCN2* gene, AD inheritance, OMIM phenotype number 605635), Familial hyperaldosteronism type III (*KCNJ5* gene, AD inheritance, OMIM phenotype number 613677), and Familial hyperaldosteronism type IV (*CACNA1H* gene, AD inheritance, OMIM phenotype number 617027) (Mittal et al., 2015; Chang et al., 2022). Pseudohyperaldosteronism type IIB/Gordon syndrome (*WNK4* gene, AD inheritance, OMIM phenotype number 614491) is associated with hypercalciuria and NL (Mabillard and Sayer, 2019).

#### 3.1.9 Hyperparathyroidism and hypoparathyroidism

Genetic causes of hyperparathyroidism and hypoparathyroidism with hypercalciuria and NL and/or NC are listed in Supplementary Table S4. Genetic causes of hyperparathyroidism with NL and/or NC are Familial primary hyperparathyroidism (*CDC73* gene, AD inheritance, OMIM phenotype number 145000) and Hyperparathyroidism 4 (*GCM2* gene, AD inheritance OMIM phenotype number 617343) (Lila et al., 2012; El Allali et al., 2021). Multiple endocrine neoplasia types I (131100), IIa (171400), and IV (610755) are also associated with hyperparathyroidism and NL and/or NC (Lila et al., 2012; El Allali et al., 2021). Hypoparathyroidism, sensorineural deafness, and renal dysplasia (*GATA3* gene, AD inheritance OMIM phenotype number 146255) is associated with hypercalciuria and NC (Chenouard et al., 2013).

#### 3.1.10 Other causes of hypercalciuria

Supplementary Table S5 lists other genetic causes of hypercalciuria and NL and/or NC. This includes Susceptibility to absorptive hypercalciuria/Familial idiopathic hypercalciuria (*ADCY10* gene, AD inheritance, OMIM phenotype number 143870) (Reed et al., 2002), Hypophosphatasia (*ALPL* gene, AR and AD inheritance, OMIM phenotype numbers 241500, 241510, 146300) (Barvencik et al., 2011; Whyte et al., 2012; Rothenbuhler and Linglart, 2017), IMAGE syndrome (*CDKN1C* gene, AD inheritance, OMIM phenotype number 614732) (Balasubramanian et al., 2010), Beckwith-Wiedemann syndrome (*CDKN1C, ICR1, KCNQ1OT1* genes, AD inheritance, OMIM phenotype number 130650) (Weksberg et al., 2010), Cystic fibrosis (*CFTR* gene, AR inheritance, OMIM phenotype number 219700) (Katz et al., 1988), Obstructive azoospermia with NL (*CLDN2* gene, XLR inheritance, OMIM phenotype number 301060) (Askari et al., 2019), Amelogenesis imperfecta type IG/Enamel-renal syndrome (*FAM20A* gene, AR inheritance, OMIM phenotype number 204690) (Wang et al., 2013), Neurodevelopmental disorder with microcephaly, cataracts, and renal abnormalities (*GEMIN4* gene, AR inheritance, OMIM phenotype number 617913) (Alazami et al., 2015), Somatic mosaic McCune-Albright syndrome (*GNAS* gene, mosaic inheritance, OMIM phenotype number 174800) (Kessel et al., 1992; Kirk et al., 1999), Mitochondrial DNA depletion syndrome 6 (*MPV17* gene, AR inheritance, OMIM phenotype number 256810) (El-Hattab et al., 2018), Multiple congenital anomalies-hypotonia-seizures syndrome 3 (*PIGT* gene, AR inheritance, OMIM phenotype number 615398) (Kvarnung et al., 2013; Kohashi et al., 2018), SHORT syndrome (*PIK3R1* gene, AD inheritance, OMIM phenotype number 269880) (Dyment et al., 2013), and Idiopathic hypercalciuria (*VDR* gene, AD inheritance) (Scott et al., 1999; Halbritter et al., 2015).

### 3.2 Conditions not primarily due to hypercalciuria

A minority of genetic diseases associated with NL and NC are not primarily due to hypercalciuria, including those secondary to hyperoxaluria, cystinuria, hyperuricosuria, xanthinuria, other metabolic disorders, and multifactorial etiologies. Each of these categories will be expanded on in detail in the following subsections.

#### 3.2.1 Conditions with hyperoxaluria

Genetic causes of hyperoxaluria with NC and/or NL are discussed in greater detail below and are shown in Table 8 and Supplementary Table S6. General recommendations for children with hyperoxaluria include limiting foods high in oxalate, avoiding vitamin C supplementation, and adequate calcium intake (Copelovitch, 2012; Scoffone and Cracco, 2018).

**TABLE 8:** Genetic causes of hyperoxaluria with nephrolithiasis and/or nephrocalcinosis.

Gene	Gene product	Phenotype	OMIM phenotype number	Inheritance	Description
*AGXT*	Alanine-glyoxylate aminotransferase	Primary hyperoxaluria type I (Cochat and Rumsby, 2013)	259900	AR	Onset at infancy to adulthood of recurrent calcium oxalate NL, NC, ESKD, systemic oxalosis
*GRHPR*	Glyoxylate and hydroxypyruvate reductase	Primary hyperoxaluria type II (Cochat and Rumsby, 2013)	260000	AR	Onset in childhood with recurrent calcium oxalate NL, NC, ESKD, systemic oxalosis
*HOGA1*	4-hydroxy-2-oxoglutarate aldolase	Primary hyperoxaluria type III (Williams et al., 2012; Cochat and Rumsby, 2013; Singh et al., 2022)	613616	AR	Onset in childhood to adulthood with recurrent calcium oxalate NL, NC, CKD, rarely ESKD, without systemic oxalosis
		HOGA1 carrier (Monico et al., 2011; Pitt et al., 2015)	N/A	Carrier	May have mild hyperoxaluria or idiopathic calcium oxalate NL
*SLC26A1*	Solute carrier family 26 member 1 (PT)	Calcium oxalate NL 1 (Gee et al., 2016)	167030	AR	Hyperoxaluria, calcium oxalate NL

AR, autosomal recessive; CKD, chronic kidney disease; ESKD, end stage kidney disease; NC, nephrocalcinosis; NL, nephrolithiasis; PT, proximal tubule.

##### 3.2.1.1 AGXT gene

*AGXT* encodes for alanine-glyoxylate aminotransferase (AGT), which is a liver peroxisomal enzyme (Cochat and Rumsby, 2013). Inactivating variants in *AGXT* result in impaired metabolism of glyoxylate into glycine by AGT, leading to increased metabolism of glyoxylate by glyoxylate reductase/hydroxypyruvate reductase (GRHPR) to glycolate and by lactate dehydrogenase (LDH) into oxalate (Cochat and Rumsby, 2013). PH type I (PH1) (OMIM phenotype number 259900) accounts for approximately 80% of cases of PH (Cochat and Rumsby, 2013). This AR condition has onset in infancy to adulthood of recurrent calcium oxalate NL, NC, ESKD, and systemic oxalosis (widespread tissue deposition of calcium oxalate) (Cochat and Rumsby, 2013).

Treatments of PH1 that have been tested include substrate reduction therapy to target enzymes responsible for production of oxalate with RNA interference (RNAi) (targeting glycolate oxidase [GO] with Lumasiran, LDH with Nedosiran) and CRISPR (targeting GO, LDH) (Zabaleta et al., 2018; Martinez-Turrillas et al., 2022; Sas et al., 2022; Baum et al., 2023; Hayes et al., 2023), small molecules to prevent AGT mistargeting (Miyata et al., 2014), enhanced intestinal oxalate degradation using probiotics (Oxalobacter formigenes) or enzymes (oxalate decarboxylase) (Milliner et al., 2018; Lingeman et al., 2019; Quintero et al., 2020), and restoration of functional enzyme conformation with chaperone therapy (vitamin B6) (Fargue et al., 2013). Definitive treatment involves combined or sequential liver and kidney transplant (Loos et al., 2023).

##### 3.2.1.2 GRHPR gene

*GRHPR* encodes for glyoxylate and hydroxypyruvate reductase (GRHPR), which an enzyme expressed throughout the body, including in the liver, specifically the hepatocyte cytosol and mitochondria (Cochat and Rumsby, 2013). Inactivating variants in *GRHPR* result in impaired metabolism of glyoxylate into glycolate by GRHRP, leading to increased metabolism of glyoxylate by AGT to glycine and by LDH into oxalate (Cochat and Rumsby, 2013). PH type II (PH2) (OMIM phenotype number 260000) is an AR condition that has onset in childhood with recurrent calcium oxalate NL, NC, ESKD, and systemic oxalosis (Cochat and Rumsby, 2013).

Treatments of PH2 that have been tested include substrate reduction therapy to target enzymes responsible production for oxalate with RNA interference (RNAi) (targeting LDH with Nedosiran) and CRISPR (targeting LDH) and enhanced intestinal oxalate degradation using probiotics (Oxalobacter formigenes) or enzymes (oxalate decarboxylase) (Milliner et al., 2018; Lingeman et al., 2019; Quintero et al., 2020; Martinez-Turrillas et al., 2022; Baum et al., 2023). Treatment with combined liver and kidney transplant has been successful in individuals with PH2 (Genena et al., 2023).

##### 3.2.1.3 HOGA1 gene

*HOGA1* encodes for 4-hydroxy-2-oxoglutarate aldolase (HOGA), which is a liver mitochondrial enzyme (Cochat and Rumsby, 2013). HOGA catalyzes metabolism from 4-hydroxy-2-oxoglutarate (HOG) to glyoxylate and pyruvate (Williams et al., 2012; Singh et al., 2022). The mechanism by which variants in *HOGA1* result in PH is unclear (Williams et al., 2012; Singh et al., 2022). PH type III (PH3) (OMIM phenotype number 613616) accounts for approximately 10% of cases of PH (Williams et al., 2012; Singh et al., 2022). PH3 is an AR condition with onset in childhood to adulthood with recurrent calcium oxalate NL, NC, CKD, rarely ESKD, and without systemic oxalosis. Data suggests that carriers (heterozygotes) of inactivating variants in *HOGA1* may have mild hyperoxaluria or idiopathic calcium oxalate NL (Monico et al., 2011; Pitt et al., 2015).

Treatments of PH3 that have been tested include substrate reduction therapy to target enzymes responsible production for oxalate with RNA interference (RNAi) (targeting LDH with Nedosiran) and CRISPR (targeting LDH) and enhanced intestinal oxalate degradation using probiotics (Oxalobacter formigenes) or enzymes (oxalate decarboxylase) (Milliner et al., 2018; Lingeman et al., 2019; Quintero et al., 2020; Martinez-Turrillas et al., 2022; Goldfarb et al., 2023).

##### 3.2.1.4 SLC26A1 gene

*SLC26A1* encodes for solute carrier family 26 member 1, an electroneutral anion exchanger (sulfate-oxalate, sulfate-bicarbonate, oxalate-bicarbonate) that is expressed in the PT (Figure 1) (Gee et al., 2016). Inactivating variants in *SLC26A1* result Calcium oxalate NL 1 (OMIM phenotype number 167030), an AR condition with hyperoxaluria and calcium oxalate NL (Gee et al., 2016).

##### 3.2.1.5 Other conditions with hyperoxaluria

Peroxisome biogenesis disorder A (Zellweger) and Peroxisome biogenesis disorder B (neonatal adrenoleukodystrophy [NALD] and infantile Refsum disease [IRD]) are characterized by deficient peroxisomal assembly with a generalized loss of peroxisomal functions (van Woerden et al., 2006; Alhazmi, 2018). Children with these disorders frequently have hyperoxaluria, hypothesized to be related to reduced glyoxylate conversion into glycine by AGT with increased oxalate production by LDH (van Woerden et al., 2006; Alhazmi, 2018).

###### 3.2.1.5.1 Peroxisome biogenesis disorder A (Zellweger)

This is an AR condition associated with the absence of peroxisomes with severe neurologic dysfunction, craniofacial abnormalities, and liver dysfunction. It has been associated with hyperoxaluria with NL and NC with variants in *PEX1* (Alhazmi, 2018), likely with variants in *PEX5* (van Woerden et al., 2006), and possibly with variants in *PEX3, PEX6, PEX 10, PEX 12, PEX13, PEX14, PEX16, PEX19,* and *PEX26* (Braverman et al., 2016; Alhazmi, 2018).

###### 3.2.1.5.2 Peroxisome biogenesis disorder B (neonatal adrenoleukodystrophy [NALD] and infantile Refsum disease [IRD])

This is an AR condition generally associated with a milder phenotype than Zellweger. It has been associated with hyperoxaluria with NL and NC with variants in *PEX1* and *PEX3* (van Woerden et al., 2006; Maxit et al., 2017), likely with variants in *PEX5* (van Woerden et al., 2006), and possibly with variants in *PEX6, PEX7, PEX10, PEX11, PEX12, PEX13, PEX16,* and *PEX26* (Braverman et al., 2016; Alhazmi, 2018).

#### 3.2.2 Conditions with cystinuria

Table 9 shows genetic causes of cystinuria with NL and/or NC, which consists of Cystinuria, and Hypotonia-cystinuria syndrome.

**TABLE 9:** Genetic causes of cystinuria with nephrolithiasis and/or nephrocalcinosis.

Gene	Gene product	Phenotype	OMIM phenotype number	Inheritance	Description
2p21/P*REPL* and *SLC31*	Prolyl endopeptidase like and Solute carrier family 3 member 1 (PT)	Hypotonia-cystinuria syndrome	606407	AR	Homozygous deletion of *PREPL* and neighboring *SLC3A1* result in hypotonia, cystinuria, cystine NL
*SLC31*	Solute carrier family 3 member 1 (PT)	Cystinuria type A (Font-Llitjós et al., 2005; Barbosa et al., 2012)	220100	AD/AR	Impaired renal reabsorption of cystine and its low solubility causes NL, resulting in obstructive uropathy, pyelonephritis, rarely ESKD
*SLC79*	Solute carrier family 7 member 9 (PT)	Cystinuria type B (Dello Strologo et al., 2002; Barbosa et al., 2012)	220100	AD/AR	Impaired renal reabsorption of cystine and its low solubility causes NL, resulting in obstructive uropathy, pyelonephritis, rarely ESKD
*SLC3A1 and SLC79*	Solute carrier family 3 member 1 (PT) and Solute carrier family 7 member 9 (PT)	Cystinuria (Font-Llitjós et al., 2005; Barbosa et al., 2012; Rhodes et al., 2015)	N/A	DR/TA	Impaired renal reabsorption of cystine and its low solubility causes NL, resulting in obstructive uropathy, pyelonephritis, rarely ESKD

AD, autosomal dominant; AR, autosomal recessive; DR, digenic recessive; ESKD, end stage kidney disease; NC, nephrocalcinosis; NL, nephrolithiasis; PT, proximal tubule; TA, triallelic.

##### 3.2.2.1 Cystinuria

This condition results in impaired renal reabsorption of cystine. Cystine’s low solubility causes the formation of NL, resulting in obstructive uropathy, pyelonephritis, and rarely ESKD (Barbosa et al., 2012). Cystinuria results from variants in *SLC3A1* and *SLC7A9*, which encode for solute carrier family 3 member 1 and solute carrier family 7 member 9, respectively, both of which are expressed in the PT (Figure 1). This condition has been associated with one or two pathogenic variants in either gene (AD/AR, OMIM phenotype number 220100 for *SLC3A1* (Font-Llitjós et al., 2005), OMIM phenotype number 220100 for *SLC7A9* (Dello Strologo et al., 2002)), one pathogenic variant in each gene (DR) (Font-Llitjós et al., 2005), or two pathogenic variants in one gene with one pathogenic variant in the other gene (triallelic inheritance) (Rhodes et al., 2015).

Mainstays in therapy are urinary dilution with hyperhydration, decreased cystine excretion through low sodium diet and low protein (methionine) diet in adolescents and adults of <0.8 g protein per day, increased urinary cystine solubility by alkalinizing the urine to pH of 7.5 with potassium citrate, and increased urinary cystine solubility by conversion of cystine to cysteine (if cystine excretion is >3 mmol per day) with chelation agents such as D-penicillamine and tiopronin (Knoll et al., 2005). Tolvaptan has been shown to decrease urinary cystine concentrations by increasing diuresis (de Boer et al., 2012). Captopril and Bucillamine are other drugs thought to increasing urinary cystine solubility by conversion of cystine to cysteine, but the data is uncertain (Knoll et al., 2005; Moussa et al., 2020). L-cystine dimethyl ester (L-CDME) and L-cystine methyl ester (L-CME) are being studied as cystine crystal growth inhibitors and alpha-lipoic acid is being studied as a drug to increase urinary cystine solubility (Rimer et al., 2010; Zee et al., 2017).

##### 3.2.2.2 Hypotonia-cystinuria syndrome

Chromosome 2p21 contains the following genes: PREPL and SLC3A1, which encode for prolyl endopeptidase like and solute carrier family 3 member 1, respectively (Jaeken et al., 2006). PREPL and SLC3A1 are expressed in the PT (Jaeken et al., 2006). Homozygous deletion of both PREPL and the neighboring gene SLC3A1 result in Hypotonia-cystinuria syndrome (AR inheritance, OMIM phenotype number 606407) with hypotonia, cystinuria, and cystine NL (Jaeken et al., 2006).

#### 3.2.3 Purine metabolism disorders

**FIGURE 2:**
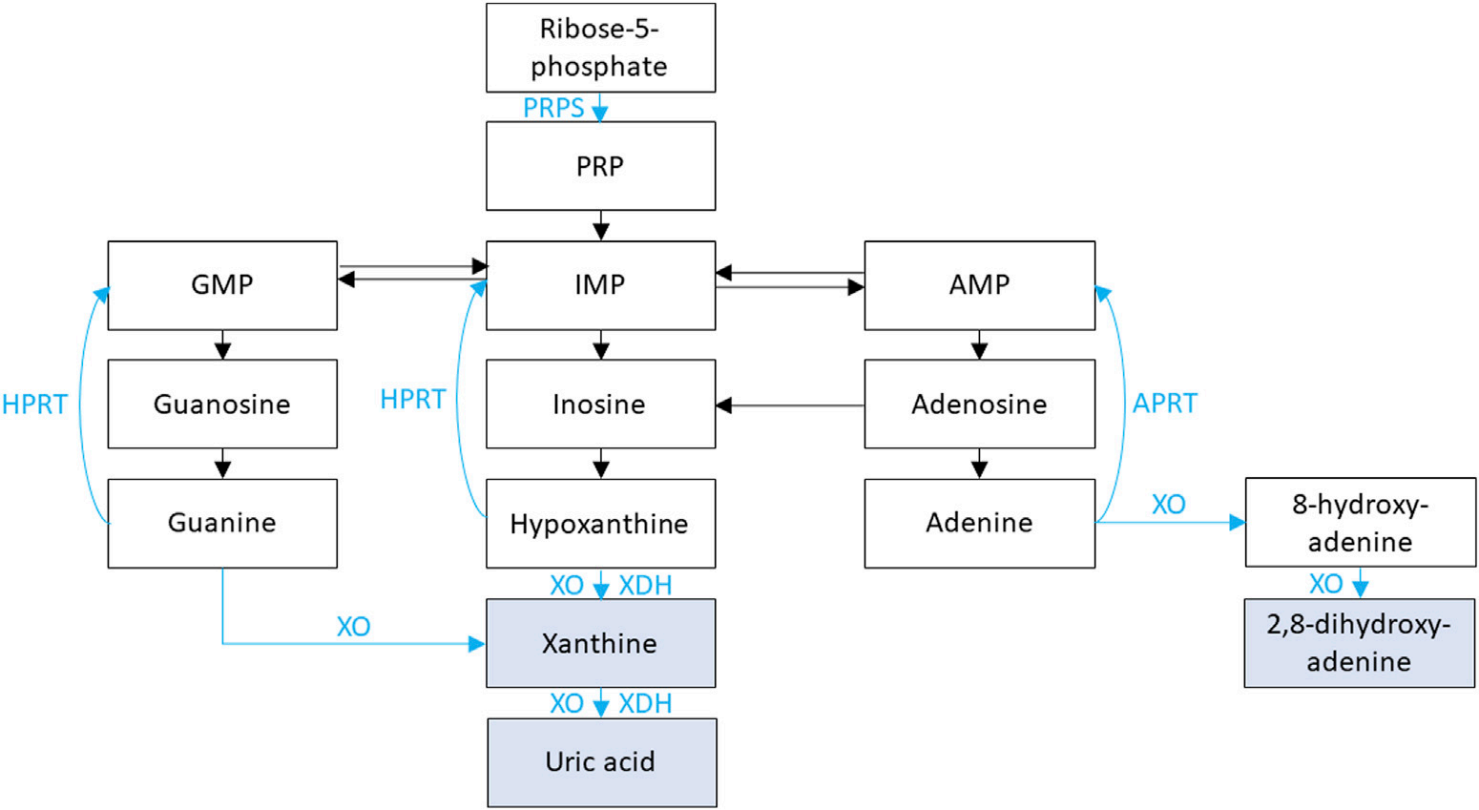
Purine metabolism is shown. Xanthine oxidase (XO) and xanthine dehydrogenase (XDH) facilitate the oxidation and reduction, respectively, of hypoxanthine to xanthine and of xanthine to uric acid. XO is also responsible for the oxidation of guanine to xanthine, the oxidation of adenine to 8-hydroxy-adenine, and the oxidation of 8-hydroxy-adenine to 2,8-dihydroxy-adenine. Phosphoribosyl pyrophosphate synthetase (PRPS) facilitates the conversion of adenosine triphosphate (ATP) and ribose-5-phosphate into phosphoribosyl pyrophosphate (PRP). PRP is subsequently converted to inosine monophosphate (IMP) then hypoxanthine. Hypoxanthine-guanine phosphoribosyl transferase (HPRT) is responsible for converting hypoxanthine IMP and guanine to guanosine monophosphate (GMP). Adenine phosphoribosyltransferase (APRT) is responsible for converting adenine to adenosine monophosphate (AMP).

Genetic causes of purine metabolism disorders with NL and/or NC are shown in Table 10. Purine metabolism is illustrated in Figure 2. The genes responsible for conditions with hyperuricosuria are *HPRT1, PRPS1, SLC22A12,* and *SLC2A9.* In addition, variation in the *ZNF365* gene has been associated susceptibility to uric acid NL (complex inheritance, OMIM phenotype number 605990) (Gianfrancesco et al., 2003). Genetic causes of xanthinuria with NL and/or NC consist of Xanthinuria (*XDH* and *MOCOS* genes) and Molybdenum cofactor deficiency (*MOCS1* and *MOCS2* genes). Molybdenum cofactor deficiency C (*GPHN* gene, OMIM phenotype number 615501) has not been associated with reports of NL or NC and will not be discussed. Treatment of these conditions generally includes hyperhydration and low purine diet (Scoffone and Cracco, 2018).

**TABLE 10:** Genetic causes of purine metabolism disorders associated with nephrolithiasis and/or nephrocalcinosis.

Gene	Gene product	Phenotype	OMIM phenotype number	Inheritance	Description
Hyperuricosuria					
*HPRT1*	Hypoxanthine-guanine phosphoribosyl transferase	Lesch-Nyhan syndrome (Jinnah et al., 2013)	300322	XLR	ID, spastic CP, choreoathetosis, self-destruction, hyperuricemia, gout, uric acid NL
		HPRT-related hyperuricemia (Jinnah et al., 2013)	300323	XLR	Hyperuricemia, gout, uric acid NL
		Female HPRT1 carrier (Fu et al., 2014)	N/A	Female carrier	Usually asymptomatic, may have HPRT-related hyperuricemia, Lesch-Nyhan syndrome
*PRPS1*	Phosphoribosyl pyrophosphate synthetase (PRPS)	PRPS-related gout/PRPS superactivity (Jinnah et al., 2013)	300661	XLR	SNHL, neurologic issues, gout, hyperuricemia, NL
		PRPS1 female carrier (Zikánová et al., 2018)	N/A	Female carrier	May have gout, hyperuricemia, NL
*SLC22A12*	Solute carrier family 22 member 12, urate-anion transporter (URAT1) (PT)	Renal hypouricemia (Enomoto et al., 2002; Tanaka et al., 2003)	220150	AR	Hypouricemia, hyperuricosuria, uric acid NL, may have exercise-induced AKF
		SLC22A12 carrier (Kawamura et al., 2020)	N/A	Carrier	May develop hypouricemia, hyperuricosuria, uric acid NL
*SLC2A9*	Solute carrier family 2 member 9, voltage-driven urate transporter/facilitated glucose transporter (GLUT9) (PT)	Renal hypouricemia 2 (Anzai et al., 2008; Androvitsanea et al., 2015)	612076	AD/AR	Hyperuricosuria, hypouricemia, uric acid NL, may have exercise-induced AKF
*ZNF365*	Zinc finger protein 365	Susceptibility to uric acid NL (Gianfrancesco et al., 2003)	605990	Complex	Susceptibility to uric acid NL
Xanthinuria					
*XDH*	Xanthine dehydrogenase	Xanthinuria type I (Arikyants et al., 2007)	278300	AR	Hypouricemia, hypouricosuria, increased hypoxanthine/xanthine production, hypoxanthinuria, xanthinuria. Some with xanthine NL, ESKD
*MOCOS*	Molybdenum cofactor sulfurase	Xanthinuria type II (Sedda et al., 2021)	603592	AR	Hypouricemia, hypouricosuria, increased hypoxanthine/xanthine production, hypoxanthinuria, xanthinuria. Some develop xanthine NL, ESKD, myositis
*MOCS1*	Molybdenum cofactor synthesis 1	Molybdenum cofactor deficiency A (Zaki et al., 2016)	252150	AR	Onset in infancy, poor feeding, seizures, severe psychomotor retardation, hypouricemia, hypouricosuria, increased sulfite/xanthine production, urinary excretion of sulfite/xanthine, xanthine NL
*MOCS2*	Molybdenum cofactor synthesis 2	Molybdenum cofactor deficiency B (Karunakar et al., 2020; Lee et al., 2021; Johannes et al., 2022)	252160	AR	Onset in infancy, poor feeding, seizures, severe psychomotor retardation, hypouricemia, hypouricosuria, increased sulfite/xanthine production, urinary excretion of sulfite/xanthine, rarely xanthine NL
Urinary 2,8-dihydroxyadenine (DHA)					
*APRT*	Adenine phosphoribosyl-transferase	Adenine phosphoribosyl-transferase deficiency (Li et al., 2019)	614723	AR	Accumulation of insoluble purine DHA, crystalluria, NL, sometime ESKD

AD, autosomal dominant; AKF, acute kidney failure; AR, autosomal recessive; CP, cerebral palsy; DHA, 2,8-dihydroxyadenine ESKD, end stage kidney disease; ID, intellectual disability; MCDK, multicystic dysplastic kidney; NL, nephrolithiasis; PT, proximal tubule; SNHL, sensorineural hearing loss; SNHL, sensorineural hearing loss; XLR, X-linked recessive.

##### 3.2.3.1 Hyperuricosuria

###### 3.2.3.1.1 HPRT1 gene

*HPRT1* encodes for hypoxanthine-guanine phosphoribosyl transferase (HPRT), which plays an important role in purine nucleotide metabolism (Figure 2) (Jinnah et al., 2013). During normal purine metabolism, xanthine oxidase (XO) facilitates the oxidation of purines hypoxanthine and guanine to xanthine and oxidation of the xanthine to uric acid (Figure 2) (Jinnah et al., 2013). HPRT is responsible for converting the purine hypoxanthine to the purine nucleotide inosine monophosphate (IMP) and the purine guanine to the purine nucleotide guanosine monophosphate (GMP) (Jinnah et al., 2013). Conversion of purines to purine nucleotides by HPRT therefore results in decreased uric acid production. Therefore, inactivating variants in *HPRT1* result in increased production of purines hypoxanthine and guanine and oxidation by XO to xanthine then uric acid with resultant hyperuricemia (Jinnah et al., 2013). Inactivating variants in *HPRT1* are associated with a spectrum of conditions depending on degree of HPRT dysfunction.

Treatment for hyperuricemia in these conditions has included hyperhydration, xanthine oxidase inhibitors (allopurinol, febuxostat), recombinant urate oxidases (rasburicase), and urinary alkalinization (Torres et al., 2012; Madeo et al., 2019). Allopurinol is considered first line treatment, but caution must be used to avoid xanthine NL that can result from excessive doses (Torres et al., 2012; Madeo et al., 2019). Lesch-Nyhan syndrome (OMIM phenotype number 300322) is an XLR condition due to an inactivating variant in *HPRT1,* resulting in hyperuricemia with subsequent intellectual disability, involuntary movements, self-injurious behavior, gout, and uric acid NL (Madeo et al., 2019). HPRT-related hyperuricemia (OMIM phenotype number 300323) is an XLR condition due to an inactivating variant in *HPRT1,* resulting in hyperuricemia with subsequent gout and uric acid NL (Madeo et al., 2019). Data suggests that female carriers of inactivating variants in *HPRT1* are usually asymptomatic but may have HPRT-related hyperuricemia or Lesch-Nyhan syndrome (Fu et al., 2014).

###### 3.2.3.1.2 PRPS1 gene

*PRPS1* encodes for phosphoribosyl pyrophosphate synthetase (PRPS), which plays an important role in purine nucleotide synthesis (Figure 2) (Jinnah et al., 2013). During normal purine metabolism, PRPS facilitates the conversion of adenosine triphosphate (ATP) and ribose-5-phosphate into phosphoribosyl pyrophosphate (PRP) (Jinnah et al., 2013). PRP is subsequently converted to the purine nucleotides IMP then hypoxanthine, and XO facilitates the oxidation of hypoxanthine to xanthine and conversion of xanthine to uric acid (Jinnah et al., 2013). Conversion of ribose-5-phosphate to PRP by PRPS therefore results in increased uric acid production. Therefore, activating variants in *PRPS1* result in increased production PRPS, IMP, hypoxanthine, xanthine, and finally uric acid (Jinnah et al., 2013). Treatment for hyperuricemia in these conditions is like that of conditions associated with *HPRT1* inactivating variants. PRPS-related gout/PRPS superactivity (OMIM phenotype number 300661) is an XLR condition due to an activating variant in *PRPS1,* resulting in hyperuricemia with sensorineural hearing loss, neurologic issues gout, and NL (Jinnah et al., 2013). Data suggests that female carriers of inactivating variants in *PRPS1* may have gout, hyperuricemia, and NL (Zikánová et al., 2018).

###### 3.2.3.1.3 SLC22A12 gene

*SLC22A12* encodes for solute carrier family 22 member 12, which is a urate-anion transporter (URAT1) expressed in the PT responsible for luminal/apical uric acid reuptake and is important in the regulation of blood urate levels (Figure 1) (Enomoto et al., 2002). Renal hypouricemia (OMIM phenotype number 220150) is an AR condition due to inactivating variants in *SLC22A1*, which lead to decreased urate transport in the PT, resulting in hypouricemia, hyperuricosuria, and uric acid NL (Enomoto et al., 2002). This condition may be associated with severe complications such as exercise-induced acute kidney failure (Tanaka et al., 2003). Patients with this condition have been successfully treated with urinary alkalinization (Hisatome et al., 1993). Data suggests that carriers (heterozygotes) of inactivating variants in *SLC22A12* may develop hypouricemia, hyperuricosuria, and uric acid NL (Kawamura et al., 2020).

###### 3.2.3.1.4 SLC2A9 gene

*SLC2A9* encodes for solute carrier family 2 member 9, which is a voltage-driven urate transporter and facilitated glucose transporter (GLUT9) expressed in the PT responsible for basolateral acid reuptake and is important in the regulation of blood urate levels (Figure 1) (Anzai et al., 2008). Renal hypouricemia 2 (OMIM phenotype number 612076) is an AD/AR condition due to inactivating variants in *SLC2A9*, resulting in impaired renal urate reabsorption with hyperuricosuria and subsequent hypouricemia and uric acid NL (Anzai et al., 2008). This condition may be associated with severe complications such as exercise-induced acute kidney failure (Androvitsanea et al., 2015). Patients with this condition have been successfully treated with urinary alkalinization (Hisatome et al., 1993).

##### 3.2.3.2 Xanthinuria

###### 3.2.3.2.1 XDH gene

*XDH* encodes for xanthine dehydrogenase (XDH) (Arikyants et al., 2007). During normal purine metabolism, XDH facilitates the conversion of hypoxanthine to xanthine and from xanthine to uric acid via reduction of NAD^+^ to NADH (Figure 2) (Arikyants et al., 2007). Xanthinuria type I (OMIM phenotype number 278300) is an AR condition due inactivating variants in *XDH*, resulting in hypouricemia with hypouricosuria, increased hypoxanthine production with hypoxanthinuria, and increased xanthine production with xanthinuria (Arikyants et al., 2007). Some individuals develop xanthine NL and/or ESKD (Arikyants et al., 2007). In addition to hyperhydration and low purine diet, inhibitors of xanthine crystallization have been tested successfully *in vitro* (Sedda et al., 2021).

###### 3.2.3.2.2 MOCOS gene

*MOCOS* encodes for molybdenum cofactor sulfurase (MOCOS), which is required to activate XDH and aldehyde oxidase 1 (AOX1) (Sedda et al., 2021). During normal purine metabolism, XDH facilitates the conversion of hypoxanthine to xanthine and from xanthine to uric acid via reduction of NAD^+^ to NADH (Arikyants et al., 2007). The physiologic relevance of AOX1 is uncertain (Sedda et al., 2021). Xanthinuria type II (OMIM phenotype number 603592) is an AR condition due inactivating variants in *MOCOS*, resulting in hypouricemia with hypouricosuria, increased hypoxanthine production with hypoxanthinuria, and increased xanthine production with xanthinuria (Sedda et al., 2021). Some individuals develop xanthine NL, ESKD, and/or myositis (Sedda et al., 2021). Treatment is the same as for Xanthinuria type I (Sedda et al., 2021).

###### 3.2.3.2.3 MOCS1 gene

*MOCS1* encodes for molybdenum cofactor synthesis 1 (MOCS1), which is responsible for the conversion from guanosine triphosphate (GTP) to cyclic pyranopterin monophosphate (cPMP), the first step in the synthesis of molybdenum cofactor (MOCO) (Reiss and Hahnewald, 2011; Johannes et al., 2022). MOCO is required to activate XDH and sulfite oxidase (SUOX) (Reiss and Hahnewald, 2011). XDH facilitates the conversion of hypoxanthine to xanthine and from xanthine to uric acid via reduction of NAD^+^ to NADH and SUOX facilitates the oxidative degradation of sulfur-containing amino acids (Arikyants et al., 2007; Zaki et al., 2016).

Molybdenum cofactor deficiency A (OMIM phenotype number 252150) is an AR condition due inactivating variants in *MOCS1*, resulting in disease onset in infancy with poor feeding, intractable seizures, severe psychomotor retardation, hypouricemia with hypouricosuria, increased sulfite production with urinary excretion of sulfite, and increased xanthine production with xanthinuria and xanthine NL (Zaki et al., 2016; Johannes et al., 2022). Treatment with synthetic cPMP (Fisdenopterin) has been shown to be effective in this condition (Farrell et al., 2021).

###### 3.2.3.2.4 MOCS2 gene

*MOCS2* encodes for molybdenum cofactor synthesis 2, which is responsible for the conversion from cPMP to molybdopterin (MPT), the second step in the synthesis of molybdenum cofactor (MOCO) (Lee et al., 2021; Johannes et al., 2022). Molybdenum cofactor deficiency B (OMIM phenotype number 252160) is an AR condition due inactivating variants in *MOCS2*, resulting in disease onset in infancy with poor feeding, intractable seizures, severe psychomotor retardation, hypouricemia with hypouricosuria, increased sulfite production with urinary excretion of sulfite, and increased xanthine production with xanthinuria and rarely xanthine NL (Karunakar et al., 2020; Lee et al., 2021; Johannes et al., 2022). There is currently no effective therapy for this condition (Johannes et al., 2022).

##### 3.2.3.3 Urinary 2,8-dihydroxyadenine (DHA)

###### 3.2.3.3.1 APRT gene

*APRT* encodes for adenine phosphoribosyltransferase (APRT), which plays an important role in purine nucleotide metabolism (Figure 2) (Li et al., 2019). APRT is responsible for converting the purine nucleotide adenine to adenosine monophosphate (AMP), which subsequently is converted to the purine nucleoside adenosine, then to the nucleoside inosine (Li et al., 2019). Insosine is then converted to the purine hypoxanthine and XO facilitates the oxidation of hypoxanthine to xanthine and xanthine to uric acid (Figure 2) (Li et al., 2019; Johannes et al., 2022).

APRT deficiency (OMIM phenotype number 614723) is an AR condition due inactivating variants in *APRT*, resulting in inability of adenine to be converted to AMP (Li et al., 2019). XO then facilitates the oxidation of adenine to 8-hydroxyadenine then 2,8-hydroxyadenine (DHA), an insoluble purine that accumulates in the kidney with crystalluria, NL, and sometime ESKD (Li et al., 2019). Treatment of APRT deficiency involves low purine diet to limit DHA production and xanthine oxidase inhibitors (allopurinol and/or febuxostat) to reduce conversion of adenine to DHA (Li et al., 2019).

#### 3.2.4 Other metabolic disorders

Supplementary Table S7 shows other genetic metabolic disorders with NL and/or NC. The conditions are 3-methylglutaconic aciduria type VIIB (*CLPB* gene, AR inheritance, OMIM phenotype number 616271) (Kanabus et al., 2015), Congenital disorder of glycosylation with defective fucosylation 1 (*FUT8* gene, AR inheritance, OMIM phenotype number 618005) (Ng et al., 2018), Glycogen storage disease type 1A (*G6PC* gene, AR inheritance, OMIM phenotype number 232200) (Weinstein et al., 2001), Alkaptonuria (*HGD* gene, AR inheritance, OMIM phenotype number 203500) (Phornphutkul et al., 2002), 5-oxoprolinase deficiency (*OPLAH* gene, AD/AR inheritance, OMIM phenotype number 260005) (Larsson et al., 1981), Dicarboxylic aminoaciduria (*SLC1A1* gene, AR inheritance, OMIM phenotype number 222730) (Bailey et al., 2011), Hyperglycinuria (*SLC36A2* gene, AD inheritance, OMIM phenotype number 138500) (De Vries et al., 1957), and Hartnup disorder (*SLC6A19* gene, AR inheritance OMIM phenotype number 234500) (Simoni et al., 2007).

#### 3.2.5 Conditions with multifactorial etiologies

##### 3.2.5.1 Disorders of inorganic pyrophosphate

Genetic disorders of inorganic pyrophosphate (PPi) with NL and/or NC are shown in Table 11. The genes responsible are *ENPP1* and *ABCC6.*

**TABLE 11:** Genetic disorders of inorganic pyrophosphate with nephrolithiasis and/or nephrocalcinosis.

Gene	Gene product	Phenotype	OMIM phenotype number	Inheritance	Description
*ABCC6*	ATP binding cassette subfamily C member 6 (PT)	Generalized arterial calcification of infancy 2 (Nitschke et al., 2012; Favre et al., 2017)	614473	AR	Deficiency of PPi, calcification of internal elastic lamina of muscular arteries, cortical NC
		Pseudoxanthoma elasticum (Vasudevan et al., 2010; Legrand et al., 2017)	264800	AR	Deficiency of PPi, accumulation of mineralized and fragmented elastic fibers in skin, vascular walls, eye Bruch membrane, NC, NL
*ENPP1*	Ectonucleotide pyrophosphatase/phosphodiesterase 1 (PT)	Generalized arterial calcification of infancy 1 (Nitschke et al., 2012; Ferreira et al., 2021a)	208000	AR	Deficiency of PPi, calcification of internal elastic lamina of muscular arteries, cortical NC
		AR hypophosphatemic rickets 2 (Lorenz-Depiereux et al., 2010; Ferreira et al., 2021b)	613312	AR	Deficiency of PPi, hypophosphatemic rickets, hyperphosphaturia, NC in multiple cases

AR, autosomal recessive; NC, nephrocalcinosis; NL, nephrolithiasis; PPi, inorganic pyrophosphate; PT, proximal tubule.

###### 3.2.5.1.1 ENPP1 gene

*ENPP1* encodes for ectonucleotide pyrophosphatase/phosphodiesterase 1 (ENPP1), which is responsible for generation of PPi and is expressed in the liver and the renal PT (Nitschke et al., 2012). Inactivating variants in *ENPP1* are associated with a spectrum of conditions.

Generalized arterial calcification of infancy 1 (OMIM phenotype number 208000) is an AR condition due to inactivating variants in *ENPP1*, resulting in deficiency of PPi with calcification of the internal elastic lamina of muscular arteries and stenosis due to myointimal proliferation as well as cortical NC, possibly due to ischemia (Ferreira et al., 2021a). Bisphosphonate therapy was previously thought to improve survival, but based on recent studies, this may not be the case (Ferreira et al., 2021a).

AR hypophosphatemic rickets 2 (OMIM phenotype number 613312) is an AR condition due to inactivating variants in *ENPP1*, resulting in deficiency of PPi and subsequent hypophosphatemic rickets with hyperphosphaturia as well as NC in multiple cases (Lorenz-Depiereux et al., 2010; Ferreira et al., 2021b). Treatment with calcitriol and oral phosphate improve skeletal symptoms, although they do not appear to improve bone mineral density and are associated with NC (Ferreira et al., 2021a). Enzyme replacement therapy in mice has been associated with improved bone mineral density without NC or ESKD and is currently being studied in humans (Ferreira et al., 2021a).

###### 3.2.5.1.2 ABCC6 gene

*ABCC6* encodes for ATP binding cassette subfamily C member 6, which through an unclear mechanism is thought to play an important role in physiologic inhibition of arterial calcification through production of PPi. *ABCC6* is expressed primarily in the liver and the renal PT (Favre et al., 2017). Inactivating variants in *ABCC6* are associated with a spectrum of conditions.

Generalized arterial calcification of infancy 2 (OMIM phenotype number 614473) is an AR condition due to inactivating variants in *ABCC6*, resulting in deficiency of PPi with calcification of the internal elastic lamina of muscular arteries and stenosis due to myointimal proliferation as well as cortical NC, possibly due to ischemia (Nitschke et al., 2012; Ferreira et al., 2021a). Pseudoxanthoma elasticum (OMIM phenotype number 264800) is an AR condition due to inactivating variants in *ABCC6*, resulting in deficiency of PPi with accumulation of mineralized and fragmented elastic fibers in the skin, vascular walls, and Bruch membrane in the eye, as well as NC and NL (Vasudevan et al., 2010; Legrand et al., 2017). As with ENNP1 variants, bisphosphonate therapy may not be effective (Kawai et al., 2022).

##### 3.2.5.2 Polycystic kidney disease

Genetic disorders with polycystic kidney disease (PKD) with NL and/or NC are shown in Supplementary Table S8, which consist of AR PKD (*PKDH1* gene, OMIM phenotype number 263200) and AD PKD including AD PKD type 1 (*PKD1* gene, OMIM phenotype number 173900), AD PKD type 2 (*PKD2* gene, OMIM phenotype number 613095), AD PKD type 3 (*GANAB* gene, OMIM phenotype number 600666), AD PKD type 6 with or without polycystic liver disease (*DNAJB11* gene, OMIM phenotype number 618061), and AD PKD type 7 (*ALG5* gene, OMIM phenotype number 620056). AR PKD is associated with multifactorial NL, NC, and medullary sponge kidney (Adeva et al., 2006). AD PKD is associated with NL (usually uric acid or calcium oxalate), abnormal transport of ammonium, low urine pH, hypocitraturia, and sometimes a distal RTA (Torres et al., 1993).

##### 3.2.5.3 Other disorders with multifactorial etiologies

Other genetic disorders with multifactorial etiologies of NL and/or NC are shown in Supplementary Table S9. The associated genes for these disorders are *CLDN10* (Klar et al., 2017), *EMC10* (Shao et al., 2021; Kaiyrzhanov et al., 2022), *HSD11B2* (Lu et al., 2022), *PAX2* (Bower et al., 2012), *STRADA* (Puffenberger et al., 2007; Bi et al., 2016; Nelson et al., 2018), and *ZNF687* (Rendina et al., 2020).

### 3.3 Conditions with possible association

Supplementary Table S10 shows a list of genetic disorders possibly associated with NL and/or NC. Variants or deletions in the following genes or genetic locations are associated with 1–2 cases of NL and/or NC: 19q13.11 (Caubit et al., 2016), *AGPAT2* (Haghighi et al., 2016), *AMMECR1* (Andreoletti et al., 2017), *ATIC* (Ramond et al., 2020), *ATP6V1E1* (Alazami et al., 2016), *BSCL2* (Haghighi et al., 2016), *CHST14* (Dundar et al., 2001), *FGF23* (Chefetz et al., 2005), *GAD1* (Neuray et al., 2020), *GNB2* (Tan et al., 2022), *IFIH1* (Buers et al., 2017), *MTM1* (Herman et al., 1999), *MYL9* (Kandler et al., 2020), *ROR2* (Tufan et al., 2005), *SLC45A1* (Srour et al., 2017), *SRCAP* (White et al., 2010), and *TMEM67* (Lee et al., 2017).

Variants in the following genes are tested for using a number of commercially available gene panels, but upon review, no cases of NL and/or NC were confirmed: *GALNT3* (Hyperphosphatemic familial tumoral calcinosis 1, AR inheritance, OMIM phenotype number 211900) and *GNA11* (AD hypocalcemia 2, AD inheritance, OMIM phenotype number 615361). For the genetic condition Developmental and epileptic encephalopathy 41 (*SLC1A2* gene, AD inheritance, OMIM phenotype number 617105), OMIM states there is an association with NC, but on review, no cases were able to be confirmed.

### 3.4 Genome-wide association studies

There have been several genome wide association studies (GWAS) of adult populations on multiple continents (North America, Europe, Asia) and in multiple countries (USA, Iceland, Netherlands, England, Japan) that have been summarized below (Thorleifsson et al., 2009; Gudbjartsson et al., 2010; Urabe et al., 2012; Oddsson et al., 2015; Howles et al., 2019; Ware et al., 2019; Curry et al., 2020). Although the results of these adult GWAS are important, there have not been any GWAS in children with NL or NC and it is unclear what the relevance of these findings are in the pediatric population.

Variants in *TRPM6,* which encodes for transient receptor potential melastatin 6 and mediates the renal and intestinal transport of magnesium, have been associated with urinary magnesium excretion (Ware et al., 2019). Variants in *CYP24A1* associated with urinary calcium excretion, vitamin D metabolism, serum calcium levels, and recurrent nephrolithiasis (Howles et al., 2019; Ware et al., 2019). Loci in *DGKD*, *DGKH*, *WDR72*, *GPIC1*, and *BCR* were found to influence signaling of CaSR (Howles et al., 2019). *DGKD* and *DGKH* encode diacylglycerol kinase, which induce CaSR-mediated intracellular calcium release (Howles et al., 2019). *GIPC1* encodes G-protein alpha-interacting protein C-terminus-interacting protein 1, which is thought to play a role in sustained intracellular CaSR signaling through clathrin-mediated endocytosis (Howles et al., 2019). *BCR* encodes breakpost cluster region, whose activation is induced by CaSR ligand binding (Howles et al., 2019).

Variants in *CLDN14, ALPL*, *CASR*, *CLDN2, SLC34A1*, *AQP1*, as well as *DGKH* have been significantly associated with NL (Thorleifsson et al., 2009; Urabe et al., 2012; Oddsson et al., 2015; Curry et al., 2020). *CLDN14* regulates paracellular permeability in the kidney epithelial tight junctions (Thorleifsson et al., 2009). *ALPL* encodes an alkaline phosphatase that is widely expressed, including in the renal PT (Oddsson et al., 2015). *CLDN2* encodes for a paracellular cation channel that mediates calcium reabsorption primarily in the PT (Curry et al., 2020). *AQP1* encodes aquaporin 1, a water channel present in the PT, thin descending loop of Henle, and vasa recta responsible for urinary concentration and water reabsorption (Urabe et al., 2012). Variants in *SLC34A1* and *TRPV5* have been associated with recurrent NL and variants in *UMOD* have been associated with both CKD and a decreased risk of NL (Gudbjartsson et al., 2010; Oddsson et al., 2015). *TRPV5* encodes an epithelial calcium channel at the apical membrane of the distal tubule that facilitates renal calcium transport, stimulated by PTH and 1,25-dihydroxy-vitamin D (Oddsson et al., 2015). *UMOD* encodes for uromodulin, the most abundant protein in the urine of mammals, and regulates endocytosis of *TRPV5* (Gudbjartsson et al., 2010).

## 4 General prevention strategies

As a reminder, aside from specific interventions mentioned above, general dietary interventions for children with NL include proper hydration, low sodium diet, maintaining adequate calcium intake, adequate but not excessive protein intake, avoidance of sugar-sweetened beverages, and a diet rich in fruits and vegetables (Copelovitch, 2012; Ferraro et al., 2013; Escribano et al., 2014; Scoffone and Cracco, 2018). However, much of this is based on adult studies and more studies are necessary in children with NL and NC. Hydration, ideally with water, of 1.5–2 L/m^2^/day is recommended to help reduce lithogenic factor concentration (Copelovitch, 2012; Scoffone and Cracco, 2018). Studies in adults with hypercalciuria have suggested that a low sodium intake, normal calcium intake, and low protein intake reduce NL recurrence and urinary calcium excretion (Escribano et al., 2014). However, it is important that growing children receive adequate recommended daily intake of calcium and protein to grow and develop appropriately, and therefore it is recommended that children ingest adequate calcium and adequate but not excessive amounts of protein (Copelovitch, 2012; Scoffone and Cracco, 2018). In adults, sugar-sweetened beverage intake has been associated with increased risk of NL (Ferraro et al., 2013). A diet rich in fruits and vegetables is recommended as they are a rich source of potassium and citrate, generally considered to be inhibitors of NL formation (Copelovitch, 2012; Scoffone and Cracco, 2018).

## 5 Discussion

The etiology of NL and NC is complex and includes environmental as well as genetic factors. As genetic testing has become more advanced, efficient, and readily available, several polygenic traits and monogenic disorders have been implicated in NL and NC. The discovery of these genes and study of these genes has greatly expanded our knowledge of the renal tubules and their channels, transporters, and receptors. However, further studies are necessary, especially in children, to better be able to provide individualized and evidence-based treatments, including the use of precision-medicine approaches.
