# Supplementary material for: Review of childhood genetic nephrolithiasis and nephrocalcinosis
Source: Front Genet. 2024 Mar 28;15:1381174. doi: 10.3389/fgene.2024.1381174 (PMC11007102; doi:10.3389/fgene.2024.1381174)
Supplement: Supplementary file 1 [file Table1.docx]

Supplementary Material

# Supplementary Figures and Tables

## Supplementary table S1. Genetic causes of secondary hypercalcemia and hypocalcemia with hypercalciuria, nephrolithiasis and/or nephrocalcinosis.

| **Gene** | **Gene product** | **Phenotype** | **OMIM phenotype number** | **Inheritance** | **Description** |
| --- | --- | --- | --- | --- | --- |
| 7q11.23 | N/A | Williams-Beuren syndrome | 194050 | AD | Facial dysmorphism, cardiovascular issues, social problems, hypertension, syndromal infantile hypercalcemia, NC |
| *PIK3C2A* | Phosphatidylinositol-4-phosphate 3-kinase catalytic subunit type 2 alpha | Oculoskeleto-dental syndrome (1) | [618440](https://www.omim.org/entry/618440) | AR | Congenital cataract, short stature, skeletal anomalies, dysmorphic facies, dental anomalies, developmental delay, stroke, hearing loss, glaucoma, hypercalcemia, hypercalciuria, NC (3 cases) |
| ? *PCSK1* | ? Proprotein convertase, subtilisin/kexin type 1 | Blue diaper syndrome (2, 3) | [211000](https://www.omim.org/entry/211000?search=Blue%20diaper%20syndrome&highlight=blue%20diaper%20syndrome%20syndromic) | ? AR,  ? XLR | Disorder of intestinal tryptophan transport, bluish colored diaper from incanduria, hypercalcemia, NC |
| *SI* | Sucrase-isomaltase | Congenital sucrase-isomaltase deficiency (4, 5) | [222900](https://www.omim.org/entry/222900) | AR | Absence of sucrase and most of maltase digestive activity, isomaltase activity absent to normal, unabsorbed disaccharides with osmotic-fermatative diarrhea, vomiting, flatulence, abdominal pain, cases with NL and NC (possibly due to hypercalcemia with dehydration) |
| *SLC5A1* | Solute carrier family 5 (sodium/glucose cotransporter) member 1 | Glucose/galactose malabsorption (6) | [606824](https://www.omim.org/entry/606824) | AR | Defect in glucose/ galactose intestinal transport, neonatal watery diarrhea, dehydration, hypercalcemia, hypercalciuria, NC, NL |

AD, autosomal dominant; AR, autosomal recessive; NC, nephrocalcinosis; NL, nephrolithiasis; XLR, X-linked recessive.

## Supplementary table S2. Genetic causes of secondary proximal tubulopathy with hypercalciuria and nephrolithiasis and/or nephrocalcinosis.

| **Gene** | **Gene product** | **Phenotype** | **OMIM phenotype number** | **Inheritance** | **Description** |
| --- | --- | --- | --- | --- | --- |
| *ALDOB* | Fructose-bisphosphate aldolase B (PT) | Hereditary fructose intolerance (7, 8) | [229600](https://www.omim.org/entry/229600) | AR | Recurrent vomiting, abdominal pain, hypoglycemia, liver failure, poor growth, possible proximal RTA, NC in 2 cases, NL in 1 case |
| *ATP7B* | Copper-transporting ATPase beta polypeptide | Wilson disease (9) | [277900](https://www.omim.org/entry/277900) | AR | Intracellular hepatic copper build-up, hepatic and neurologic abnormalities, hypercalciuria, NC, NL |
| *CTNS* | Cystinosin, lysosomal cystine transporter (PT) | Nephropathic cystinosis (10) | [219800](https://www.omim.org/entry/219800) | AR | Lysosomal storage disorder, accumulation of cystine in cells, poor growth, hypophosphatemic/ calcipenic rickets, ESKD, Fanconi renotubular syndrome, hypercalciuria, hyperphosphaturia, NC, NL |
| *FAH* | Fumaryl-acetoacetate hydrolase (PT) | Tyrosinemia type 1 (11) | [276700](https://www.omim.org/entry/276700) | AR | Deficiency of fumaryl-acetoacetase (tyrosine degradation enzyme), liver disease, Fanconi renotubular syndrome, hypophosphatemic rickets, NC |
| *LCT* | Lactase-phlorizin hydrolase | Congenital lactase deficiency (12) | [223000](https://www.omim.org/entry/223000) | AR | Vomiting, diarrhea, failure to thrive, dehydration, disacchariduria, RTA, amino aciduria, metabolic acidosis, hypercalcemia, hypercalciuria, NC |
| *RRM2B* | Ribonucleotide reductase regulatory TP53 inducible subunit M2B | Mitochondrial DNA depletion syndrome 8A (encephalo-myopathic type with renal tubulopathy) (13) | [612075](https://www.omim.org/entry/612075) | AR | Neonatal hypotonia, lactic acidosis, neurologic deterioration, proximal tubulopathy, NC |
| *TRNT1* | Mitochondrial CCA-adding tRNA nucleotidyl-transferase | Sideroblastic anemia with B-cell immune-deficiency, periodic fevers, developmental delay (14) | [616084](https://www.omim.org/entry/616084) | AR | SNHL, retinitis pigmentosa, cardiomyopathy, Fanconi renotubular syndrome, NC, few cases of NL |
| *VIPAS39* | VPS33b-interacting protein | Arthrogryposis, renal dysfunction, and cholestasis 2 (15) | [613404](https://www.omim.org/entry/613404) | AR | Joint contractures, cholestasis, failure to thrive, ichthyosis, Fanconi renotubular syndrome, NC |
| *VPS33B* | Homolog of yeast vacuolar protein sorting 33 b | Arthrogryposis, renal dysfunction, and cholestasis 1 (15) | [208085](https://www.omim.org/entry/208085) | AR | Joint contractures, cholestasis, failure to thrive, ichthyosis, Fanconi renotubular syndrome, NC |

AR, autosomal recessive; ESKD, end stage kidney disease; NC, nephrocalcinosis; NL, nephrolithiasis; PT, proximal tubule; RTA, renal tubular acidosis; SNHL, sensorineural hearing loss.

## Supplementary table S3. Genetic causes of hyperaldosteronism and pseudohyperaldosteronism with hypercalciuria and nephrolithiasis and/or nephrocalcinosis.

| **Gene** | **Gene product** | **Phenotype** | **OMIM phenotype number** | **Inheritance** | **Description** |
| --- | --- | --- | --- | --- | --- |
| *CACNA1H* | Calcium voltage-gated channel subunit alpha-1 H | Familial HA type IV (16, 17) | [617027](https://www.omim.org/entry/617027) | AD | Hypertension, HA, hypokalemia, possibly NC, NL |
| *CLCN2* | Chloride voltage-gated channel 2 | Familial HA type II (16, 17) | [605635](https://www.omim.org/entry/605635) | AD | Hypertension, HA, hypokalemia, possibly NC, NL |
| *CYP11B1* | Cytochrome P450 family 11 subfamily B member 1 | Glucocorticoid-remediable aldosteronism / Familial HA type I (16, 17) | [103900](https://www.omim.org/entry/103900) | AD | Hypertension, low plasma renin activity, HA, abnormal adrenal steroid production, responsive to dexamethasone treatment, possibly NC, NL |
| *KCNJ5* | Potassium inwardly rectifying channel subfamily J member 5 | Familial HA type III (16, 17) | [613677](https://www.omim.org/entry/613677) | AD | HA, hypertension, high levels of hybrid steroids 18-oxocortisol and 18-hydroxycortisol, require adrenalectomy to control hypertension, possibly NL, NC |
| *WNK4* | WNK lysine deficient protein kinase 4 (DCT, CD) | Pseudo-hyperaldosteronism type IIB / Gordon syndrome (18) | [614491](https://www.omim.org/entry/614491) | AD | Hypocalcemia, decreased bone mineral density, hypercalciuria, NL |

AD, autosomal dominant; CD, collecting duct; DCT, distal convoluted tubule; HA, hyperaldosteronism; NC, nephrocalcinosis; NL, nephrolithiasis.

## Supplementary table S4. Genetic causes of hyperparathyroidism and hypoparathyroidism with hypercalciuria and nephrolithiasis and/or nephrocalcinosis.

| **Gene** | **Gene product** | **Phenotype** | **OMIM phenotype number** | **Inheritance** | **Description** |
| --- | --- | --- | --- | --- | --- |
| *CDC73* | Cell division cycle 73 | Familial primary hyperparathyroidism (19, 20) | 145000 | AD | Overproduction of PTH, hypercalcemia, hypercalciuria, NL, NC |
| *GCM2* | Glial cells missing transcription factor 2 | Hyperparathyroidism 4 (19, 20) | [617343](https://www.omim.org/entry/617343) | AD | Overproduction of PTH, hypercalcemia, hypercalciuria, NL, NC |
| *GATA3* | GATA-binding protein 3 | Hypoparathyroidism, sensorineural deafness, renal dysplasia (21) | [146255](https://www.omim.org/entry/146255) | AD | Hypoparathyroidism, SNHL, may have CAKUT, NS, hematuria, proteinuria, CKD, ESKD, RTA, hypercalciuria, NC |
| *MEN1* | Menin 1 | MEN type I (19, 20) | [131100](https://www.omim.org/entry/131100) | AD | Overproduction of PTH, hypercalcemia, hypercalciuria, NL, NC |
| *RET* | Rearranged during transfection protooncogene | MEN type IIA (19, 20) | [171400](https://www.omim.org/entry/171400) | AD | Overproduction of PTH, hypercalcemia, hypercalciuria, NL, NC |
| *CDKN1B* | Cyclin-dependent kinase inhibitor 1B | MEN type IV (19, 20) | [610755](https://www.omim.org/entry/610755) | AD | Overproduction of PTH, hypercalcemia, hypercalciuria, NL, NC |

AD, autosomal dominant; CAKUT, congenital anomalies of the kidney and urinary tract; CKD, chronic kidney disease; ESKD, end stage kidney disease; MEN, multiple endocrine neoplasia; NC, nephrocalcinosis; NL, nephrolithiasis; NS, nephrotic syndrome; PTH, parathyroid hormone; RTA, renal tubular acidosis; SNHL, sensorineural hearing loss.

## Supplementary table S5. Other genetic causes of hypercalciuria and nephrolithiasis and/or nephrocalcinosis.

| **Gene** | **Gene product** | **Phenotype** | **OMIM phenotype number** | **Inheritance** | **Description** |
| --- | --- | --- | --- | --- | --- |
| *ADCY10* | Adenylyl cyclase 10 | Susceptibility to absorptive hypercalciuria / Familial idiopathic hypercalciuria (22) | [143870](https://www.omim.org/entry/143870) | AD | Hypercalciuria, recurrent NL |
| *ALPL* | Bio-mineralization associated alkaline phosphatase (PT) | Infantile Hypophosphatasia (23) | [241500](https://www.omim.org/entry/241500) | AR | First 6 months of life PPi inhibits bone mineralization, failure to thrive, hyporexia, muscle weakness, delayed motor development, rickets, hypercalciuria, NC, sometimes NL |
|  |  | Childhood Hypophosphatasia (24) | [241510](https://www.omim.org/entry/241510) | AR | After 6 months old PPi inhibits bone mineralization, rickets, short stature, muscle weakness, delayed motor development, may have hypercalciuria, NC |
|  |  | Adult Hypophosphatasia (25) | [146300](https://www.omim.org/entry/146300) | AD/AR | In adults PPi inhibits bone mineralization, osteomalacia, pseudofractures, fractures, muscle/joint pain, cases of NC (uncertain if due to treatment) |
| *CDKN1C* | Cyclin-dependent kinase inhibitor 1C | IMAGE Syndrome (26) | [614732](https://www.omim.org/entry/614732) | AD | IUGR, metaphyseal dysplasia, congenital adrenal hypoplasia, genital anomalies, craniosynostosis, cleft palate, scoliosis, hypercalciuria, hypocalcemia, NC |
| *CDKN1C* | Cyclin-dependent kinase inhibitor 1c | Beckwith-Wiedemann syndrome (27) | [130650](https://www.omim.org/entry/130650) | AD | Rapid growth in first few years, hemihypertrophy, macroglossia, hypoglycemia, abdominal wall defects, visceromegaly, Wilms tumor, hepatoblastoma, CAKUT, hypercalciuria, NC, NL |
| *ICR1* | H19/IGF2-imprinting control region |  |  |  |  |
| *KCNQ1OT1* | KCNQ1-opposite strand / antisense transcript 1 |  |  |  |  |
| *CFTR* | Cystic fibrosis transmembrane conductance regulator | Cystic fibrosis (28) | [219700](https://www.omim.org/entry/219700) | AR | COPD, exocrine pancreatic insufficiency, high NaCl in sweat, hypercalciuria, NC |
| *CLDN2* | Claudin-2, integral membrane tight junction protein (PT) | Obstructive azoospermia with NL (29) | [301060](https://www.omim.org/entry/301060) | XLR | Male infertility, hypercalciuria, NL |
| *FAM20A* | Family with sequence similarity 20 member A | Amelogenesis imperfecta type IG / Enamel-renal syndrome (30) | [204690](https://www.omim.org/entry/204690) | AR | Hypoplastic enamel, pulp stones, delayed/failed eruption of secondary dentition, gingival overgrowth, NC of unclear etiology |
| *GEMIN4* | Gem nuclear organelle associated protein 4 | Neuro-developmental disorder with microcephaly, cataracts, renal abnormalities (31) | [617913](https://www.omim.org/entry/617913) | AR | Complex severe neurodevelopmental disorder, microcephaly, cataracts, GERD, seizures, hyporeflexia, CAKUT, hypertension, tubulopathy, NL, NC |
| *GNAS* | GNAS complex locus | Somatic mosaic McCune-Albright syndrome (32, 33) | [174800](https://www.omim.org/entry/174800) | Mosaic | Postzygotic somatic mutation early in development, monoclonal population of mutated cells, polyostotic fibrous dysplasia, cafe-au-lait spots, peripheral precocious puberty, thyrotoxicosis, pituitary gigantism, Cushing syndrome, NC mostly related to hypercalciuria |
| *MPV17* | Mitochondrial inner membrane protein MPV17 | Mitochondrial DNA depletion syndrome 6 (34) | [256810](https://www.omim.org/entry/256810) | AR | Infantile progressive liver failure, progressive neurologic issues, eye abnormalities, hypoparathyroidism, tubulopathy, NC |
| *PIGT* | Phosphatidyl-inositol glycan anchor biosynthesis class T | Multiple congenital anomalies-hypotonia-seizures syndrome 3 (35, 36) | [615398](https://www.omim.org/entry/615398) | AR | Neonatal hypotonia, seizures, dysmorphic features, variable congenital anomalies including CAKUT, hypercalciuria with NC (5 cases) |
| *PIK3R1* | Phospho-inositide-3-kinase regulatory subunit 1 | SHORT syndrome (37) | [269880](https://www.omim.org/entry/269880) | AD | Short stature, joint hyperextensibility, hernias, eye anomalies, teething delay, dysmorphic facies, partial lipodystrophy, insulin resistance, hearing deficits, hypercalciuria with NC |
| *VDR* | 1,25-dihydroxy-vitamin D3 receptor | Idiopathic hypercalciuria (38, 39) | N/A | AD | Hypercalciuria, NL |

AD, autosomal dominant; AR, autosomal recessive; CAKUT, congenital anomalies of the kidney and urinary tract; COPD, chronic obstructive pulmonary disease; GERD, gastroesophageal reflux disease; IUGR, Intrauterine growth restriction; NaCl, sodium chloride; NC, nephrocalcinosis; NL, nephrolithiasis; PPi, inorganic pyrophosphate; PT, proximal tubule; XLR, X-linked recessive.

## Supplementary table S6. Other genetic causes of hyperoxaluria with nephrolithiasis and/or nephrocalcinosis.

| **Gene** | **Gene product** | **Phenotype** | **OMIM phenotype number** | **Inheritance** | **Description** |
| --- | --- | --- | --- | --- | --- |
| *PEX1 (40)* | Peroxisomal biogenesis factor 1 | Peroxisome biogenesis disorder A (Zellweger) | [214100](https://www.omim.org/entry/214100) | AR | Absence of peroxisomes, severe neurologic dysfunction, craniofacial abnormalities, liver dysfunction, hyperoxaluria with NL and NC with *PEX1*, likely *PEX5*, possibly *PEX3, PEX6, PEX 10, PEX 12, PEX13, PEX14, PEX16, PEX19, PEX26* |
| *PEX3 (41)* | Peroxisomal biogenesis factor 3 |  | [614882](https://www.omim.org/entry/614882) |  |  |
| *PEX5 (42)* | Peroxisomal biogenesis factor 5 |  | [214110](https://www.omim.org/entry/214110) |  |  |
| *PEX6 (40)* | Peroxisomal biogenesis factor 6 |  | [614862](https://www.omim.org/entry/620152) |  |  |
| *PEX10 (41)* | Peroxisomal biogenesis factor 10 |  | [614870](https://www.omim.org/entry/614870) |  |  |
| *PEX12 (41)* | Peroxisomal biogenesis factor 12 |  | [614859](https://www.omim.org/entry/614859) |  |  |
| *PEX13 (41)* | Peroxisomal biogenesis factor 13 |  | [614883](https://www.omim.org/entry/614883) |  |  |
| *PEX14 (40)* | Peroxisomal biogenesis factor 14 |  | [614887](https://www.omim.org/entry/614887) |  |  |
| *PEX16 (41)* | Peroxisomal biogenesis factor 16 |  | [614876](https://www.omim.org/entry/614876) |  |  |
| *PEX19 (40)* | Peroxisomal biogenesis factor 19 |  | [614886](https://www.omim.org/entry/614886) |  |  |
| *PEX26 (41)* | Peroxisomal biogenesis factor 26 |  | [614872](https://www.omim.org/entry/614872) |  |  |
| *PEX1 (42)* | Peroxisomal biogenesis factor 1 | Peroxisome biogenesis disorder B (NALD and IRD) | [601539](https://www.omim.org/entry/601539) | AR | Milder phenotype than Zellweger, hyperoxaluria with NL and NC with *PEX1* and *PEX3*, likely *PEX5*, possibly *PEX6, PEX7, PEX10, PEX11, PEX12, PEX13, PEX16, PEX26* |
| *PEX3 (43)* | Peroxisomal biogenesis factor 3 |  | [617370](https://www.omim.org/entry/617370) |  |  |
| *PEX5 (42)* | Peroxisomal biogenesis factor 5 |  | [202370](https://www.omim.org/entry/202370) |  |  |
| *PEX6 (41)* | Peroxisomal biogenesis factor 6 |  | [614863](https://www.omim.org/entry/612075) |  |  |
| *PEX7 (41)* | Peroxisomal biogenesis factor 7 |  | [614879](https://www.omim.org/entry/614879) |  |  |
| *PEX10 (41)* | Peroxisomal biogenesis factor 10 |  | [614871](https://www.omim.org/entry/614871) |  |  |
| *PEX11 (41)* | Peroxisomal biogenesis factor 11 |  | [614920](https://www.omim.org/entry/614920) |  |  |
| *PEX12 (40)* | Peroxisomal biogenesis factor 12 |  | [266510](https://www.omim.org/entry/266510) |  |  |
| *PEX13 (40)* | Peroxisomal biogenesis factor 13 |  | [614885](https://www.omim.org/entry/614885) |  |  |
| *PEX16 (40)* | Peroxisomal biogenesis factor 16 |  | [614877](https://www.omim.org/entry/614877) |  |  |
| *PEX26 (40)* | Peroxisomal biogenesis factor 26 |  | [614873](https://www.omim.org/entry/614873) |  |  |

AR, autosomal recessive; IRD, infantile Refsum disease; NALD, neonatal adrenoleukodystrophy; NC, nephrocalcinosis; NL, nephrolithiasis.

## Supplementary table S7. Other genetic metabolic disorders with nephrolithiasis and/or nephrocalcinosis.

| **Gene** | **Gene product** | **Phenotype** | **OMIM phenotype number** | **Inheritance** | **Description** |
| --- | --- | --- | --- | --- | --- |
| *CLPB* | Caseinolytic mitochondrial matrix peptidase chaperone subunit B | 3-methyl-glutaconic aciduria type VIIB (44) | [616271](https://www.omim.org/entry/616271) | AR | Cataracts, neurologic deficits, neutropenia, NC in at least 2 cases |
| *FUT8* | Fucosyl-transferase 8 | Congenital disorder of glycosylation with defective fucosylation 1 (45) | [618005](https://www.omim.org/entry/618005) | AR | Poor growth, failure to thrive, hypotonia, skeletal anomalies, delayed psychomotor development, ID, NC |
| *G6PC* | Glucose-6-phosphatase catalytic subunit 1 (PT) | Glycogen storage disease type 1A (46) | [232200](https://www.omim.org/entry/232200) | AR | Glycogen accumulation, severe hypoglycemia, hepatomegaly, growth retardation, delayed puberty, lactic acidemia, hyperlipidemia, hyperuricemia, gout, nephropathy, NL, hypercalciuria, NC |
| *HGD* | Homogentisate 1,2-dioxygenase (PT) | Alkaptonuria (47) | [203500](https://www.omim.org/entry/203500) | AR | Homogentisic acid accumulation, darkened urine, connective tissue pigmentation, joint/spine arthritis, cardiac valves destruction, NL |
| *OPLAH* | 5-oxoprolinase, ATP-hydrolysing | 5-oxoprolinase deficiency (48) | 260005 | AD/AR | Inborn error of glutathione metabolism, excessive excretion of 5-oxo-L-proline, NL |
| *SLC1A1* | Solute carrier family 1 member 1 (PT) | Dicarboxylic aminoaciduria (49) | [222730](https://www.omim.org/entry/222730) | AR | Incomplete glomerular anionic amino acid reabsorption, elevated urinary glutamate/aspartate, NL |
| *SLC36A2* | Solute carrier family 36 member 2 (PT) | Hyper-glycinuria (50) | [138500](https://www.omim.org/entry/138500) | AD | Glycinuria, oxalate NL |
| *SLC6A19* | Solute carrier family 6 member 19 (PT) | Hartnup disorder (51) | [234500](https://www.omim.org/entry/234500) | AR | Aminoaciduria, pellagra-like light-sensitive rash, cerebellar ataxia, emotional instability, hyperuricosuria, uric acid NL |

AD, autosomal dominant; AR, autosomal recessive; ESKD, end stage kidney disease; ID, intellectual disability; NC, nephrocalcinosis; NL, nephrolithiasis.

## Supplementary table S8. Genetic disorders with polycystic kidney disease with nephrolithiasis and/or nephrocalcinosis.

| **Gene** | **Gene product** | **Phenotype** | **OMIM phenotype number** | **Inheritance** | **Description** |
| --- | --- | --- | --- | --- | --- |
| *ALG5* | ALG5 dolichyl-phosphate beta-glucosyltransferase | AD PKD 7 (52) | [620056](https://www.omim.org/entry/620056) | AD | NL (usually uric acid or calcium oxalate), abnormal transport of ammonium, low urine pH, hypocitraturia, some with distal RTA |
| *DNAJB11* | DnaJ heat shock protein family (Hsp40) member B11 | AD PKD 6 with or without polycystic liver disease (52) | [618061](https://www.omim.org/entry/618061) | AD | NL (usually uric acid or calcium oxalate), abnormal transport of ammonium, low urine pH, hypocitraturia, some with distal RTA |
| *GANAB* | Glucosidase II alpha subunit | AD PKD 3 (52) | [600666](https://www.omim.org/entry/600666) | AD | NL (usually uric acid or calcium oxalate), abnormal transport of ammonium, low urine pH, hypocitraturia, some with distal RTA |
| *PKD1* | Polycystin 1, transient receptor potential channel interacting | AD PKD 1 (52) | 173900 | AD | NL (usually uric acid or calcium oxalate), abnormal transport of ammonium, low urine pH, hypocitraturia, some with distal RTA |
| *PKD2* | Polycystin 2, transient receptor potential cation channel | AD PKD 2 (52) | [613095](https://www.omim.org/entry/613095) | AD | NL (usually uric acid or calcium oxalate), abnormal transport of ammonium, low urine pH, hypocitraturia, some with distal RTA |
| *PKHD1* | PKHD1 ciliary IPT domain containing fibrocystin/polyductin | AR PKD 4 with or without hepatic disease (53) | [263200](https://www.omim.org/entry/263200) | AR | Multifactorial NL, NC, medullary sponge kidney |

AD, autosomal dominant; AR, autosomal recessive; NC, nephrocalcinosis; NL, nephrolithiasis; PKD, polycystic kidney disease; RTA, renal tubular acidosis.

## Supplementary table S9. Other genetic disorders with multifactorial etiologies of nephrolithiasis and/or nephrocalcinosis.

| **Gene** | **Gene product** | **Phenotype** | **OMIM phenotype number** | **Inheritance** | **Description** |
| --- | --- | --- | --- | --- | --- |
| *CLDN10* | Claudin 10 (PT) | HELIX syndrome (54) | [617671](https://www.omim.org/entry/617671) | AR | Hypohidrosis, electrolyte imbalance, lacrimal gland dysfunction, ichthyosis, xerostomia, hypermagnesemia, abnormal renal absorption of cations, hypomagnesuria, hypocalciuria, NL |
| *EMC10* | ER membrane protein complex subunit 10 | Neuro-developmental disorder with dysmorphic facies and variable seizures (55, 56) | [619264](https://www.omim.org/entry/619264) | AR | Global developmental delay, seizures, dysmorphic facies, skeletal defects, CAKUT, NC (5 cases) |
| *HSD11B2* | Hydroxysteroid 11-beta- dehydrogenase 2 (CD) | Apparent mineralocorticoid excess (57) | [218030](https://www.omim.org/entry/218030) | AR | Decreased conversion of active cortisol to inactive cortisone, cortisol acts as a ligand for MR, sodium retention with hypernatremia, volume expansion, hypertension (low renin/aldosterone), metabolic alkalosis, hypokalemia, NC (possible chronic hypokalemia-induced interstitial nephritis) |
| *PAX2* | Paired box 2 | Papillorenal syndrome (58) | [120330](https://www.omim.org/entry/120330) | AD | CAKUT, FSGS, multiple cases of hyperuricemia, NL |
| *STRADA* | STE20 related adaptor alpha | Polyhydramnios, megalencephaly, and symptomatic epilepsy (59-61) | [611087](https://www.omim.org/entry/611087) | AR | No abnormal urine findings, NC in multiple cases, 1 case with concurrent NL |
| *ZNF687* | Zinc finger protein 687 | Paget disease of bone 6 (62) | [616833](https://www.omim.org/entry/616833) | AD | Polyostotic bone lesions primarily affecting axial skeleton, bone pain coronary artery disease, malignant giant cell tumor of bone, hearing loss, hypercalciuria, hyperoxaluria, NL |

AD, autosomal dominant; AR, autosomal recessive; CAKUT, congenital anomalies of the kidney ad urinary tract; CD, collecting duct; FSGS, focal segmental glomerulosclerosis; MR, mineralocorticoid receptor; NC, nephrocalcinosis; NL, nephrolithiasis

## Supplementary table S10. Genetic disorders possibly associated with nephrolithiasis and/or nephrocalcinosis.

| **Gene** | **Gene product** | **Phenotype** | **OMIM phenotype number** | **Inheritance** | **Description** |
| --- | --- | --- | --- | --- | --- |
| 19q13.11 | N/A | Proximal chromosome 19q13.11 deletion syndrome (63) | [617219](https://www.omim.org/entry/617219) | AD | Neurodevelopmental disorder, autism, feeding difficulties, growth retardation, kidney abnormalities (1 case with NL) |
| *AGPAT2* | 1-acylglycerol-3-phosphate O-acyltransferase 2 | Congenital generalized lipodystrophy type 1 (64) | [608594](https://www.omim.org/entry/608594) | AR | Near absence of adipose tissue, severe insulin resistance, NL (2 cases) |
| *AMMECR1* | AMMECR nuclear protein 1 | Midface hypoplasia, hearing impairment, elliptocytosis, NC (65) | [300990](https://www.omim.org/entry/300990) | XLR | 2 cases of this disease ever documented (2 half-brothers with NC, 1 with intermittent hypercalciuria) |
| *ATIC* | 5-aminoimidazole-4-carboxamide ribonucleotide formyltransferase/ IMP cyclohydrolase | AICA-ribosiduria due to ATIC deficiency (66) | [608688](https://www.omim.org/entry/608688) | AR | Global neurodevelopmental impairment, chorioretinal atrophy, growth impairment, scoliosis, coarse facies, epilepsy, aortic coarctation, chronic hepatic cytolysis, genital malformations, NC (1 case) |
| *ATP6V1E1* | V-ATPase H+ transporting V1 subunit E1 (alpha-IC, TAL, DCT) | AR cutis laxa type IIC (67) | [617402](https://www.omim.org/entry/617402) | AR | Skin wrinkling, sparse subcutaneous fat, dysmorphic progeroid facial features, hypotonia, cardiovascular involvement, NC (2 cases, brothers) |
| *BSCL2* | Seipin | Congenital generalized lipodystrophy type 2 (64) | [269700](https://www.omim.org/entry/269700) | AR | Near absence of adipose tissue, severe insulin resistance, hypertriglyceridemia, hepatic steatosis, NL (1 case) |
| *CHST14* | Carbohydrate sulfotransferase 14 | Ehlers-Danlos syndrome Musculo-contractural type 1 (68) | [601776](https://www.omim.org/entry/601776) | AR | Craniofacial dysmorphism, congenital thumb/finger contractures, clubfeet, kyphoscoliosis, hypotonia, hyperextensible thin skin, easy bruisability, atrophic scarring, wrinkled palms, joint hypermobility, ocular involvement, NL (1 case) |
| *FGF23* | Fibroblast growth factor 23 | Hyperphosphatemic familial tumoral calcinosis 2 (69) | [617993](https://www.omim.org/entry/617993) | AR | Progressive deposition of basic calcium phosphate crystals, increased renal phosphate absorption, hyperphosphatemia, NC (2 cases) |
| *GAD1* | Glutamate decarboxylase 1 | Developmental and epileptic encephalopathy 89 (70) | [619124](https://www.omim.org/entry/619124) | AR | Global developmental delay, ID, absent speech, axial hypotonia, spastic quadriparesis, seizures, joint contractures, foot deformities, dysmorphic facies, cleft palate, omphalocele, NL and NC (1 case) |
| *GNB2* | G protein subunit beta 2 | Neuro-developmental disorder with hypotonia and dysmorphic facies (71) | 619503 | AD | Global developmental delay, hypotonia, ID, dysmorphic facies, cardiac defects, joint contractures or hyperextensibility, dry skin, cryptorchidism, CAKUT, NC (1 case) |
| *IFIH1* | Interferon induced with helicase C domain 1 | Aicardi-Goutieres syndrome 7 (72) | [615846](https://www.omim.org/entry/615846) | AD | Inflammatory disorder, severe neurologic impairment, NC (1 case) |
| *MTM1* | Myotubularin 1 | X-linked centronuclear myopathy (73) | [310400](https://www.omim.org/entry/310400) | XLR | Congenital myopathy, slowly progressive weakness/wasting, pyloric stenosis, spherocytosis, gallstones, vitamin K-responsive bleeding diathesis, rapid linear growth, advanced bone age, NL (1 case), NC (1 case) |
| *MYL9* | Myosin light chain 9 | Megacystis-microcolon-intestinal hypoperistalsis syndrome 4 (74) | [619365](https://www.omim.org/entry/619365) | AR | Impaired smooth muscle contractility in bladder and intestines, bronchopulmonary dysplasia, recurrent NL (1 case) |
| *ROR2* | Receptor tyrosine kinase like orphan receptor 2 | AR Robinow syndrome 1 (75) | [268310](https://www.omim.org/entry/268310) | AR | Severe skeletal dysplasia, dysmorphic facies, short-limbed dwarfism, vertebral segmentation, genital hypoplasia, hypocitraturia with NC and NL (1 case) |
| *SLC45A1* | Solute carrier family 45 member 1 | Intellectual developmental disorder with neuropsychiatric features (76) | [617532](https://www.omim.org/entry/617532) | AR | ID, seizures, neuropsychiatric abnormalities, dysmorphic facies, NC (1 case) |
| *SRCAP* | Snf2 related CREBBP activator protein | Floating-Harbor Syndrome (77) | [136140](https://www.omim.org/entry/136140) | AD | Proportionate short stature, delayed bone age, delayed speech, dysmorphic facies, CAKUT, 2 cases of NC, 1 case with concurrent NL with hypercalciuria |
| *TMEM67* | Transmembrane protein 67 | COACH syndrome 1 (78) | [216360](https://www.omim.org/entry/216360) | AR | Cerebellar vermis hypo/aplasia, oligophrenia, ataxia, ocular coloboma, hepatic fibrosis, NL (1 case) |

AD, autosomal dominant; AR, autosomal recessive; CAKUT, congenital anomalies of the kidney and urinary tract; DCT, distal convoluted tubule; ID, intellectual disability; NC, nephrocalcinosis; NL, nephrolithiasis; TAL, thick ascending loop of Henle; XLR, X-linked recessive.

# REFERENCES

1. Tiosano D, Baris HN, Chen A, Hitzert MM, Schueler M, Gulluni F, et al. Mutations in PIK3C2A cause syndromic short stature, skeletal abnormalities, and cataracts associated with ciliary dysfunction. PLoS Genet. 2019;15(4):e1008088.

2. Drummond KN, Michael AF, Ulstrom RA, Good RA. The blue diaper syndrome: Familial hypercalcemia with nephrocalcinosis and indicanuria: A new familial disease, with definition of the metabolic abnormality. The American Journal of Medicine. 1964;37(6):928-48.

3. Distelmaier F, Herebian D, Atasever C, Beck-Woedl S, Mayatepek E, Strom TM, et al. Blue Diaper Syndrome and PCSK1 Mutations. Pediatrics. 2024;141(Supplement_5).

4. Belmont JW, Reid B, Taylor W, Baker SS, Moore WH, Morriss MC, et al. Congenital sucrase-isomaltase deficiency presenting with failure to thrive, hypercalcemia, and nephrocalcinosis. BMC Pediatr. 2002;2:4.

5. Starnes C, Welsh J. Intestinal sucrase-isomaltase deficiency and renal calculi. The New England journal of medicine. 1970;282(18).

6. Soylu ÖB, Ecevit Ç, Altınöz S, Öztürk AA, Temizkan AK, Maeda M, et al. Nephrocalcinosis in glucose-galactose malabsorption: nephrocalcinosis and proximal tubular dysfunction in a young infant with a novel mutation of SGLT1. European Journal of Pediatrics. 2008;167(12):1395-8.

7. Higgins RB, Varney JK. Dissolution of renal calculi in a case of hereditary fructose intolerance and renal tubular acidosis. J Urol. 1966;95(3):291-6.

8. Mass RE, Smith WR, Walsh JR. The association of hereditary fructose intolerance and renal tubular acidosis. Am J Med Sci. 1966;251(5):516-23.

9. Di Stefano V, Lionetti E, Rotolo N, La Rosa M, Leonardi S. Hypercalciuria and nephrocalcinosis as early feature of Wilson disease onset: description of a pediatric case and literature review. Hepat Mon. 2012;12(8):e6233.

10. Theodoropoulos DS, Shawker TH, Heinrichs C, Gahl WA. Medullary nephrocalcinosis in nephropathic cystinosis. Pediatr Nephrol. 1995;9(4):412-8.

11. Forget S, Patriquin HB, Dubois J, Lafortune M, Merouani A, Paradis K, et al. The kidney in children with tyrosinemia: sonographic, CT and biochemical findings. Pediatr Radiol. 1999;29(2):104-8.

12. Saarela T, Similä S, Koivisto M. Hypercalcemia and nephrocalcinosis in patients with congenital lactase deficiency. J Pediatr. 1995;127(6):920-3.

13. Finsterer J, Scorza FA. Renal manifestations of primary mitochondrial disorders. Biomed Rep. 2017;6(5):487-94.

14. Wiseman DH, May A, Jolles S, Connor P, Powell C, Heeney MM, et al. A novel syndrome of congenital sideroblastic anemia, B-cell immunodeficiency, periodic fevers, and developmental delay (SIFD). Blood. 2013;122(1):112-23.

15. Holme A, Hurcombe JA, Straatman-Iwanowska A, Inward CI, Gissen P, Coward RJ. Glomerular involvement in the arthrogryposis, renal dysfunction and cholestasis syndrome. Clin Kidney J. 2013;6(2):183-8.

16. Chang CK, Chang CC, Wu VC, Geng JH, Lee HY. The Relationship Between Renal Stones and Primary Aldosteronism. Front Endocrinol (Lausanne). 2022;13:828839.

17. Mittal K, Anandpara K, Dey AK, Sharma R, Thakkar H, Hira P, et al. An Association of Chronic Hyperaldosteronism with Medullary Nephrocalcinosis. Pol J Radiol. 80. Poland2015. p. 417-24.

18. Mabillard H, Sayer JA. The Molecular Genetics of Gordon Syndrome. Genes (Basel). 2019;10(12).

19. Lila AR, Sarathi V, Jagtap V, Bandgar T, Menon PS, Shah NS. Renal manifestations of primary hyperparathyroidism. Indian J Endocrinol Metab. 2012;16(2):258-62.

20. El Allali Y, Hermetet C, Bacchetta J, Amouroux C, Rothenbuhler A, Porquet-Bordes V, et al. Presenting features and molecular genetics of primary hyperparathyroidism in the paediatric population. Eur J Endocrinol. 2021;184(2):347-55.

21. Chenouard A, Isidor B, Allain-Launay E, Moreau A, Le Bideau M, Roussey G. Renal phenotypic variability in HDR syndrome: glomerular nephropathy as a novel finding. Eur J Pediatr. 2013;172(1):107-10.

22. Reed BY, Gitomer WL, Heller HJ, Hsu MC, Lemke M, Padalino P, et al. Identification and characterization of a gene with base substitutions associated with the absorptive hypercalciuria phenotype and low spinal bone density. J Clin Endocrinol Metab. 2002;87(4):1476-85.

23. Whyte MP, Greenberg CR, Salman NJ, Bober MB, McAlister WH, Wenkert D, et al. Enzyme-replacement therapy in life-threatening hypophosphatasia. N Engl J Med. 2012;366(10):904-13.

24. Rothenbuhler A, Linglart A. Hypophosphatasia in children and adolescents: clinical features and treatment. Arch Pediatr. 2017;24(5s2):5s66-5s70.

25. Barvencik F, Beil FT, Gebauer M, Busse B, Koehne T, Seitz S, et al. Skeletal mineralization defects in adult hypophosphatasia--a clinical and histological analysis. Osteoporos Int. 2011;22(10):2667-75.

26. Balasubramanian M, Sprigg A, Johnson DS. IMAGe syndrome: Case report with a previously unreported feature and review of published literature. Am J Med Genet A. 2010;152a(12):3138-42.

27. Weksberg R, Shuman C, Beckwith JB. Beckwith-Wiedemann syndrome. Eur J Hum Genet. 2010;18(1):8-14.

28. Katz SM, Krueger LJ, Falkner B. Microscopic nephrocalcinosis in cystic fibrosis. N Engl J Med. 1988;319(5):263-6.

29. Askari M, Karamzadeh R, Ansari-Pour N, Karimi-Jafari MH, Almadani N, Sadighi Gilani MA, et al. Identification of a missense variant in CLDN2 in obstructive azoospermia. J Hum Genet. 2019;64(10):1023-32.

30. Wang SK, Aref P, Hu Y, Milkovich RN, Simmer JP, El-Khateeb M, et al. FAM20A mutations can cause enamel-renal syndrome (ERS). PLoS Genet. 2013;9(2):e1003302.

31. Alazami AM, Patel N, Shamseldin HE, Anazi S, Al-Dosari MS, Alzahrani F, et al. Accelerating novel candidate gene discovery in neurogenetic disorders via whole-exome sequencing of prescreened multiplex consanguineous families. Cell Rep. 2015;10(2):148-61.

32. Kessel D, Hall CM, Shaw DG. Two unusual cases of nephrocalcinosis in infancy. Pediatr Radiol. 1992;22(6):470-1.

33. Kirk JM, Brain CE, Carson DJ, Hyde JC, Grant DB. Cushing's syndrome caused by nodular adrenal hyperplasia in children with McCune-Albright syndrome. J Pediatr. 1999;134(6):789-92.

34. El-Hattab AW, Wang J, Dai H, Almannai M, Staufner C, Alfadhel M, et al. MPV17-related mitochondrial DNA maintenance defect: New cases and review of clinical, biochemical, and molecular aspects. Hum Mutat. 2018;39(4):461-70.

35. Kohashi K, Ishiyama A, Yuasa S, Tanaka T, Miya K, Adachi Y, et al. Epileptic apnea in a patient with inherited glycosylphosphatidylinositol anchor deficiency and PIGT mutations. Brain Dev. 2018;40(1):53-7.

36. Kvarnung M, Nilsson D, Lindstrand A, Korenke GC, Chiang SC, Blennow E, et al. A novel intellectual disability syndrome caused by GPI anchor deficiency due to homozygous mutations in PIGT. J Med Genet. 2013;50(8):521-8.

37. Dyment DA, Smith AC, Alcantara D, Schwartzentruber JA, Basel-Vanagaite L, Curry CJ, et al. Mutations in PIK3R1 cause SHORT syndrome. Am J Hum Genet. 2013;93(1):158-66.

38. Halbritter J, Baum M, Hynes A, Rice S, Thwaites D, Gucev Z, et al. Fourteen monogenic genes account for 15% of nephrolithiasis/nephrocalcinosis. Journal of the American Society of Nephrology : JASN. 2015;26(3).

39. Scott P, Ouimet D, Valiquette L, Guay G, Proulx Y, Trouvé ML, et al. Suggestive evidence for a susceptibility gene near the vitamin D receptor locus in idiopathic calcium stone formation. J Am Soc Nephrol. 1999;10(5):1007-13.

40. Alhazmi HH. Renal oxalate stones in children with Zellweger spectrum disorders. Saudi J Anaesth. 12. India2018. p. 332-4.

41. Braverman NE, Raymond GV, Rizzo WB, Moser AB, Wilkinson ME, Stone EM, et al. Peroxisome biogenesis disorders in the Zellweger spectrum: An overview of current diagnosis, clinical manifestations, and treatment guidelines. Mol Genet Metab. 2016;117(3):313-21.

42. van Woerden CS, Groothoff JW, Wijburg FA, Duran M, Wanders RJ, Barth PG, et al. High incidence of hyperoxaluria in generalized peroxisomal disorders. Mol Genet Metab. 2006;88(4):346-50.

43. Maxit C, Denzler I, Marchione D, Agosta G, Koster J, Wanders RJA, et al. Novel PEX3 Gene Mutations Resulting in a Moderate Zellweger Spectrum Disorder. JIMD Rep. 2017;34:71-5.

44. Kanabus M, Shahni R, Saldanha JW, Murphy E, Plagnol V, Hoff WV, et al. Bi-allelic CLPB mutations cause cataract, renal cysts, nephrocalcinosis and 3-methylglutaconic aciduria, a novel disorder of mitochondrial protein disaggregation. J Inherit Metab Dis. 2015;38(2):211-9.

45. Ng BG, Xu G, Chandy N, Steyermark J, Shinde DN, Radtke K, et al. Biallelic Mutations in FUT8 Cause a Congenital Disorder of Glycosylation with Defective Fucosylation. Am J Hum Genet. 2018;102(1):188-95.

46. Weinstein DA, Somers MJ, Wolfsdorf JI. Decreased urinary citrate excretion in type 1a glycogen storage disease. J Pediatr. 2001;138(3):378-82.

47. Phornphutkul C, Introne WJ, Perry MB, Bernardini I, Murphey MD, Fitzpatrick DL, et al. Natural history of alkaptonuria. N Engl J Med. 2002;347(26):2111-21.

48. Larsson A, Mattsson B, Wauters EA, van Gool JD, Duran M, Wadman SK. 5-oxoprolinuria due to hereditary 5-oxoprolinase deficiency in two brothers--a new inborn error of the gamma-glutamyl cycle. Acta Paediatr Scand. 1981;70(3):301-8.

49. Bailey CG, Ryan RM, Thoeng AD, Ng C, King K, Vanslambrouck JM, et al. Loss-of-function mutations in the glutamate transporter SLC1A1 cause human dicarboxylic aminoaciduria. J Clin Invest. 2011;121(1):446-53.

50. De Vries A, Kochwa S, Lazebnik J, Frank M, Djaldetti M. Glycinuria, a hereditary disorder associated with nephrolithiasis. Am J Med. 1957;23(3):408-15.

51. Simoni RE, Gomes LN, Scalco FB, Oliveira CP, Aquino Neto FR, de Oliveira ML. Uric acid changes in urine and plasma: an effective tool in screening for purine inborn errors of metabolism and other pathological conditions. J Inherit Metab Dis. 2007;30(3):295-309.

52. Torres VE, Wilson DM, Hattery RR, Segura JW. Renal stone disease in autosomal dominant polycystic kidney disease. Am J Kidney Dis. 1993;22(4):513-9.

53. Adeva M, El-Youssef M, Rossetti S, Kamath PS, Kubly V, Consugar MB, et al. Clinical and molecular characterization defines a broadened spectrum of autosomal recessive polycystic kidney disease (ARPKD). Medicine (Baltimore). 2006;85(1):1-21.

54. Klar J, Piontek J, Milatz S, Tariq M, Jameel M, Breiderhoff T, et al. Altered paracellular cation permeability due to a rare CLDN10B variant causes anhidrosis and kidney damage. PLoS Genet. 2017;13(7):e1006897.

55. Shao DD, Straussberg R, Ahmed H, Khan A, Tian S, Hill RS, et al. A recurrent, homozygous EMC10 frameshift variant is associated with a syndrome of developmental delay with variable seizures and dysmorphic features. Genet Med. 2021;23(6):1158-62.

56. Kaiyrzhanov R, Rocca C, Suri M, Gulieva S, Zaki MS, Henig NZ, et al. Biallelic loss of EMC10 leads to mild to severe intellectual disability. Ann Clin Transl Neurol. 2022;9(7):1080-9.

57. Lu YT, Zhang D, Zhang QY, Zhou ZM, Yang KQ, Zhou XL, et al. Apparent mineralocorticoid excess: comprehensive overview of molecular genetics. J Transl Med. 2022;20(1):500.

58. Bower M, Salomon R, Allanson J, Antignac C, Benedicenti F, Benetti E, et al. Update of PAX2 mutations in renal coloboma syndrome and establishment of a locus-specific database. Hum Mutat. 2012;33(3):457-66.

59. Nelson K, Jackman C, Bell J, Shih CS, Payne K, Dlouhy S, et al. Novel Homozygous Deletion in STRADA Gene Associated With Polyhydramnios, Megalencephaly, and Epilepsy in 2 Siblings: Implications for Diagnosis and Treatment. J Child Neurol. 2018;33(14):925-9.

60. Bi W, Glass IA, Muzny DM, Gibbs RA, Eng CM, Yang Y, et al. Whole exome sequencing identifies the first STRADA point mutation in a patient with polyhydramnios, megalencephaly, and symptomatic epilepsy syndrome (PMSE). Am J Med Genet A. 2016;170(8):2181-5.

61. Puffenberger EG, Strauss KA, Ramsey KE, Craig DW, Stephan DA, Robinson DL, et al. Polyhydramnios, megalencephaly and symptomatic epilepsy caused by a homozygous 7-kilobase deletion in LYK5. Brain. 2007;130(Pt 7):1929-41.

62. Rendina D, De Filippo G, Merlotti D, Di Stefano M, Mingiano C, Giaquinto A, et al. Increased Prevalence of Nephrolithiasis and Hyperoxaluria in Paget Disease of Bone. J Clin Endocrinol Metab. 2020;105(12).

63. Caubit X, Gubellini P, Andrieux J, Roubertoux PL, Metwaly M, Jacq B, et al. TSHZ3 deletion causes an autism syndrome and defects in cortical projection neurons. Nat Genet. 2016;48(11):1359-69.

64. Haghighi A, Kavehmanesh Z, Salehzadeh F, Santos-Simarro F, Van Maldergem L, Cimbalistiene L, et al. Congenital generalized lipodystrophy: identification of novel variants and expansion of clinical spectrum. Clin Genet. 2016;89(4):434-41.

65. Andreoletti G, Seaby EG, Dewing JM, O'Kelly I, Lachlan K, Gilbert RD, et al. AMMECR1: a single point mutation causes developmental delay, midface hypoplasia and elliptocytosis. J Med Genet. 2017;54(4):269-77.

66. Ramond F, Rio M, Héron B, Imbard A, Marie S, Billiemaz K, et al. AICA-ribosiduria due to ATIC deficiency: Delineation of the phenotype with three novel cases, and long-term update on the first case. J Inherit Metab Dis. 2020;43(6):1254-64.

67. Alazami AM, Al-Qattan SM, Faqeih E, Alhashem A, Alshammari M, Alzahrani F, et al. Expanding the clinical and genetic heterogeneity of hereditary disorders of connective tissue. Hum Genet. 2016;135(5):525-40.

68. Dundar M, Kurtoglu S, Elmas B, Demiryilmaz F, Candemir Z, Ozkul Y, et al. A case with adducted thumb and club foot syndrome. Clin Dysmorphol. 2001;10(4):291-3.

69. Chefetz I, Heller R, Galli-Tsinopoulou A, Richard G, Wollnik B, Indelman M, et al. A novel homozygous missense mutation in FGF23 causes Familial Tumoral Calcinosis associated with disseminated visceral calcification. Hum Genet. 2005;118(2):261-6.

70. Neuray C, Maroofian R, Scala M, Sultan T, Pai GS, Mojarrad M, et al. Early-infantile onset epilepsy and developmental delay caused by bi-allelic GAD1 variants. Brain. 2020;143(8):2388-97.

71. Tan NB, Pagnamenta AT, Ferla MP, Gadian J, Chung BH, Chan MC, et al. Recurrent de novo missense variants in GNB2 can cause syndromic intellectual disability. J Med Genet. 2022;59(5):511-6.

72. Buers I, Rice GI, Crow YJ, Rutsch F. MDA5-Associated Neuroinflammation and the Singleton-Merten Syndrome: Two Faces of the Same Type I Interferonopathy Spectrum. J Interferon Cytokine Res. 2017;37(5):214-9.

73. Herman GE, Finegold M, Zhao W, de Gouyon B, Metzenberg A. Medical complications in long-term survivors with X-linked myotubular myopathy. J Pediatr. 1999;134(2):206-14.

74. Kandler JL, Sklirou E, Woerner A, Walsh L, Cox E, Xue Y. Compound heterozygous loss of function variants in MYL9 in a child with megacystis-microcolon-intestinal hypoperistalsis syndrome. Mol Genet Genomic Med. 2020;8(11):e1516.

75. Tufan F, Cefle K, Türkmen S, Türkmen A, Zorba U, Dursun M, et al. Clinical and molecular characterization of two adults with autosomal recessive Robinow syndrome. Am J Med Genet A. 2005;136(2):185-9.

76. Srour M, Shimokawa N, Hamdan FF, Nassif C, Poulin C, Al Gazali L, et al. Dysfunction of the Cerebral Glucose Transporter SLC45A1 in Individuals with Intellectual Disability and Epilepsy. Am J Hum Genet. 2017;100(5):824-30.

77. White SM, Morgan A, Da Costa A, Lacombe D, Knight SJ, Houlston R, et al. The phenotype of Floating-Harbor syndrome in 10 patients. Am J Med Genet A. 2010;152a(4):821-9.

78. Lee SH, Nam TS, Li W, Kim JH, Yoon W, Choi YD, et al. Functional validation of novel MKS3/TMEM67 mutations in COACH syndrome. Sci Rep. 2017;7(1):10222.
